# Supplementary material for: Plant death caused by inefficient induction of antiviral R-gene-mediated resistance may function as a suicidal population resistance mechanism
Source: Commun Biol. 2021 Aug 9;4:947. doi: 10.1038/s42003-021-02482-7 (PMC8352862; doi:10.1038/s42003-021-02482-7)
Supplement: Supplementary file 2 — Supplementary information [file 42003_2021_2482_MOESM2_ESM.pdf]

Supplementary Materials for

Plant death caused by inefficient induction of antiviral *R*-gene-mediated resistance may function as a suicidal population resistance mechanism

Derib A Abebe[1], Sietske van Bentum[1,2], Machi Suzuki[1], Sugihiro Ando[1], Hideki Takahashi[1], and Shuhei Miyashita[1,*]

[1] Graduate School of Agricultural Science, Tohoku University, Sendai 980-0845, Japan

[2] Department of Biology, Utrecht University, Utrecht 3584 CH, the Netherlands

[*]Corresponding author; e-mail address: shuhei.miyashita.d7@tohoku.ac.jp

This PDF file includes:

Supplementary Figs. 1 to 12: figures refered in Main text;

Supplementary Tables 1 to 5: tables refered in Main text;

Supplementary Texts 1 to 5: R scripts used in the study;

Supplementary Figs. 13 to 25: Uncropped, unedited images for Figs. and Supplementary Figs.

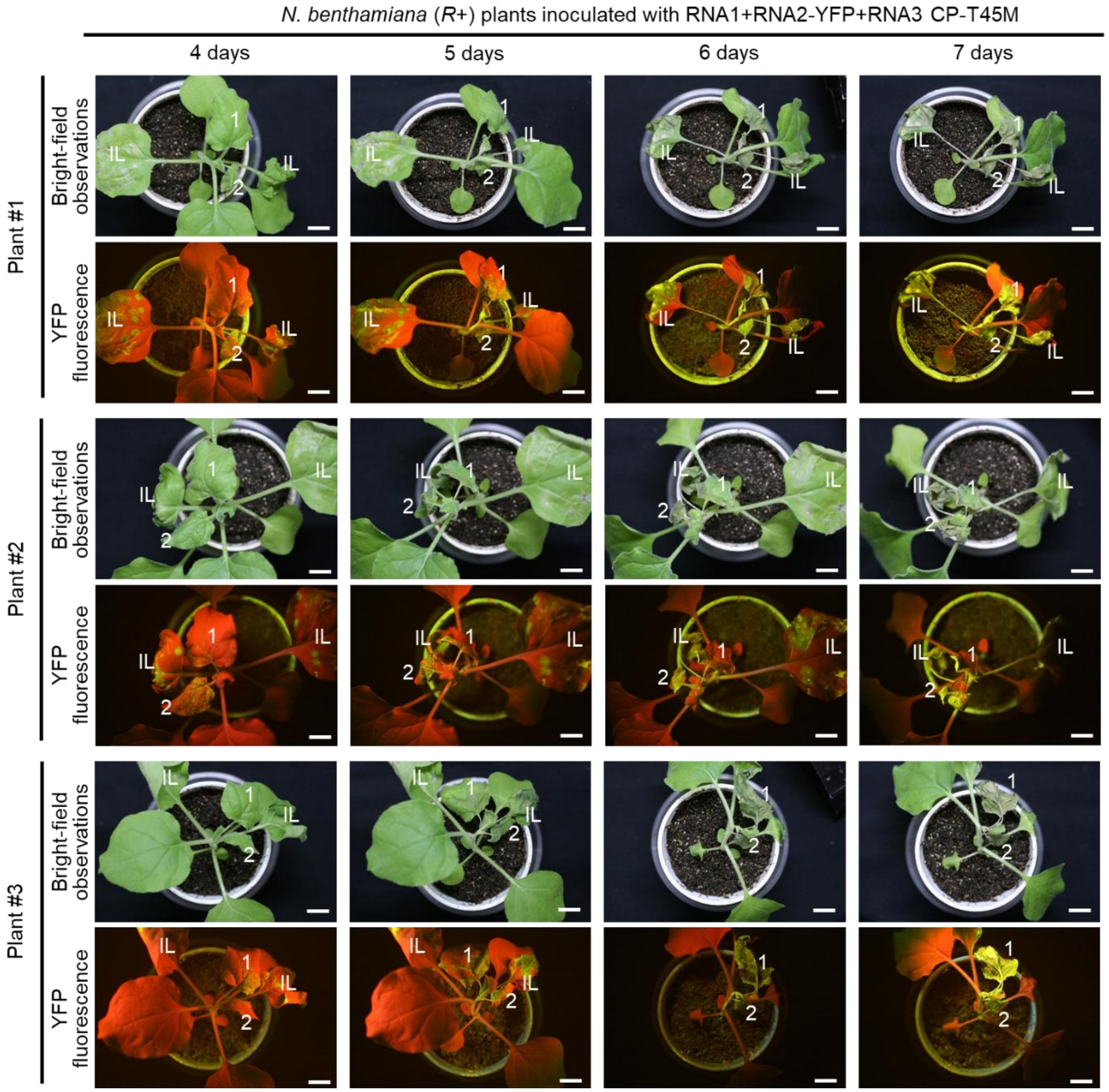

Supplementary Fig. 1. Time-course observations of *N. benthamiana* (*R*+) plants at 4 to 7 days after inoculation of RNA1, RNA2-YFP, and RNA3 CP-T45M. Images of three independent plants for bright-field and YFP-fluorescence observations are shown. IL, inoculated leaves; 1 and 2, upper uninoculated leaves that are infected by systemic movement of the virus. Uninfected regions of leaves appear red in YFP-fluorescence observations due to autofluorescence; infected regions appear yellow. Note that upper uninoculated leaves (1 and 2) first get infected and then exhibit necrosis. Scale bars =1 cm.

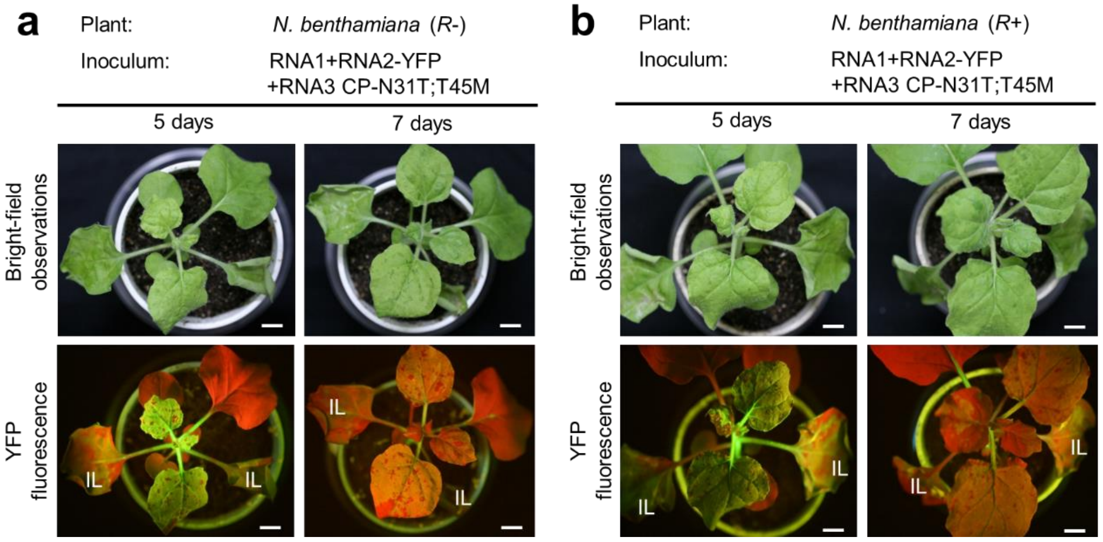

Supplementary Fig. 2. Inoculation of CP double mutant with N31T and T45M substitutions. Representative images for bright-field and YFP-fluorescence observations of a *N. benthamiana* (*R*+) plant (**a**) and a *N. benthamiana* (*R*-) plant (**b**) at 5 and 7 days after inoculation of RNA1, RNA2-YFP, and RNA3 CP-N31;T45M double mutant. IL indicate inoculated leaves. Scale bars =1 cm. Similar results were observed in more than 3 replicates of experiments.

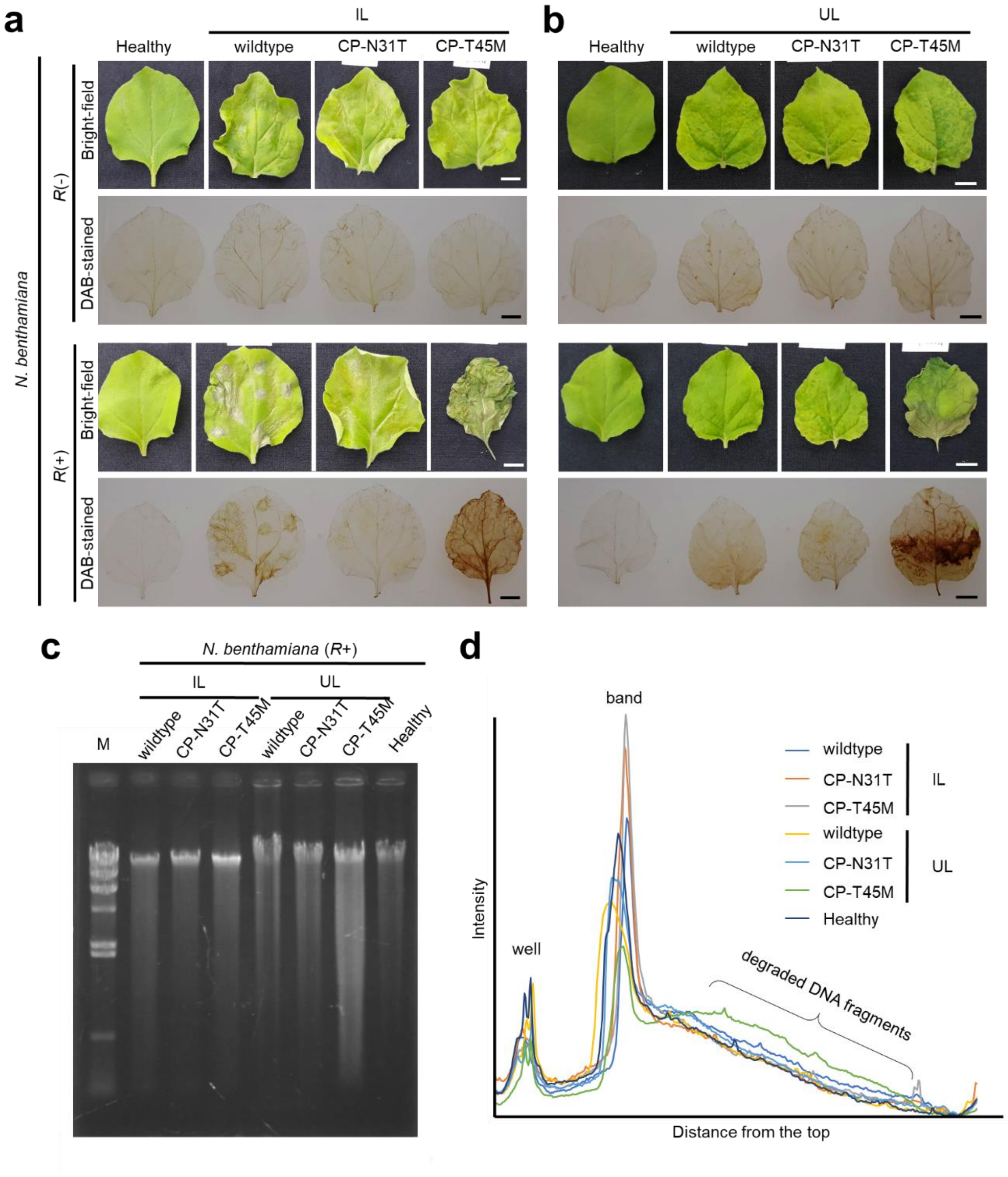

Supplementary Fig. 3. DAB staining and DNA fragmentation. Detection of H_2O_2 production by 3,3'-diaminobenzidine (DAB) staining of inoculated (**a**) and uninoculated upper (**b**) leaves of *N. benthamiana* at 5 days after inoculation of wildtype CMV and its CP variants. Scale bars = 1 cm. Note that H_2O_2 production is visualized as reddish-brown color. Similar results were observed in 3 replicates of experiments. Genomic DNA extraction of *N. benthamiana* (*R*+) leaves were also performed at 5 days after inoculation. The result of agarose gel electrophoresis (**c**) and quantified signal intensities after normalization against total intensities (**d**) are shown. Note that samples from upper leaves of CP-T45M-inoculated plants and inoculated leaves with wildtype CMV exhibit higher intensity for smaller DNA, suggesting that genomic DNAs are fragmented in these samples.

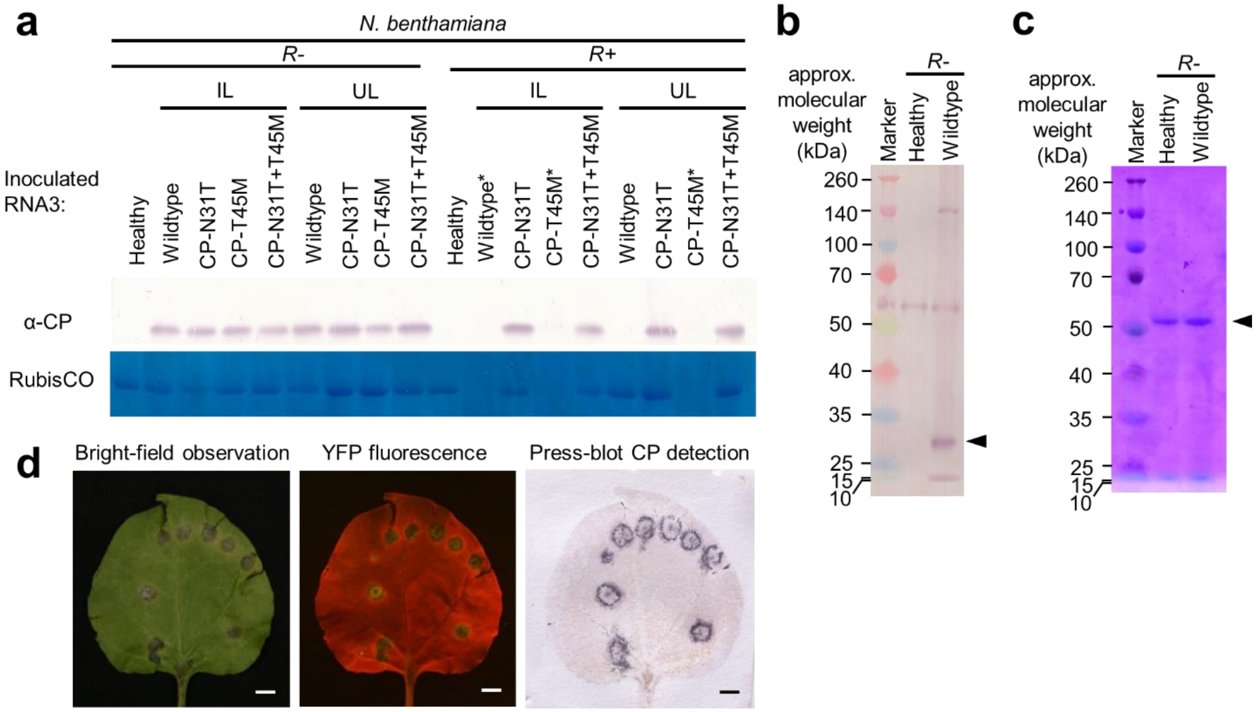

Supplementary Fig. 4. Detection of CMV(Y)-CP in inoculated and upper leaves of *N. benthamiana* (*R-*) and (*R+)* plants. a Viral CP in inoculated and uninoculated upper leaves of *N. benthamiana* (*R*-) and (*R*+) plants were detected by western blot analysis at 7 days after inoculation of wildtype RNA3 or its CP variants together with wildtype RNA1 and RNA2-YFP. Sample concentrations were normalized by sampled leaf area sizes. CBB staining image of RubisCO large subunit protein also shown. Similar results were observed in 3 replicates of experiments. Note that RubisCO large subunit protein is not detectable in necrotic leaf samples (labeled with asterisks*). **b**, **c** Examples of Western blot analysis (**b**) and CBB staining (**c**) performed together with a prestained protein size marker (Spectra™ Multicolor Broad Range Protein Ladder, Thermo Fisher Scientific, US). Samples from healthy and wildtype CMV-inoculated *N. benthamiana* (*R*-) plants were analyzed. Approximate molecular weights of the marker bands are shown in the left. Note that expected sizes of CMV CP and RubisCO large subunit are ~24 kDa and ~53 kDa, respectively, and thus the band(s) with the arrowheads were considered to be those proteins. **d** press-blot detection of viral CP accumulated in a *N. benthamiana* (*R*+) leaf at 7 days after inoculation of wildtype RNA3 with wildtype RNA1 and RNA2-YFP. Bright-field observation, YFP fluorescence observation, and immunological detection of viral CP after press blotting were shown. Scale bars = 1 cm

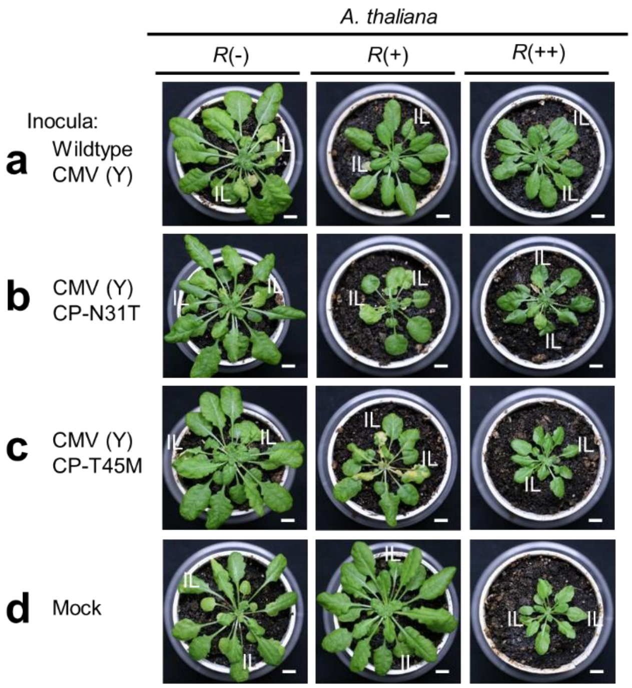

Supplementary Fig. 5. Observation of *A. thaliana* plants at 14 days after inoculation. Representative images for *A. thaliana* plants (*R*-, *R*+ and *R*++) at 14 days after inoculation with wildtype CMV(Y) (**a**), and its CP variants, CP-N31T (**b**) and CP-T45M (**c**) are shown. Mock inoculated plants also shown as negative control (**d**). IL; inoculated leaves. Scale bars=1 cm. Similar results were observed in 3 replicates of experiments.

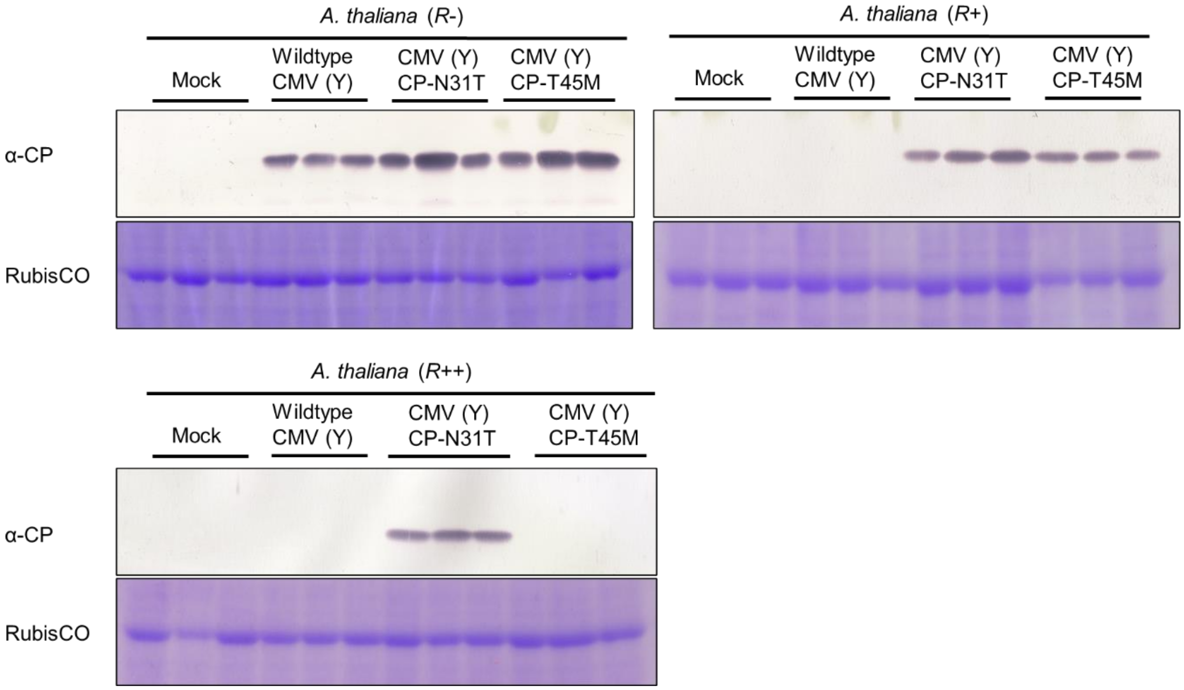

Supplementary Fig. 6. Detection of CMV (Y) CP in systemically infected upper leaves. Viral CP in upper uninoculated leaves of *A. thaliana* (*R*–), (*R*+), and (*R*++) were detected by Western blot analysis at 7 days after inoculation of WT CMV(Y) or its CP variants. Note that systemic necrosis was hardly visible in *A. thaliana* (*R*+) plants inoculated with CMV(Y) CP-T45M at this sampling time point, but subsequently became visible. CBB staining images of RubisCO protein are shown as loading controls. Three biological replicates for each combination are shown.

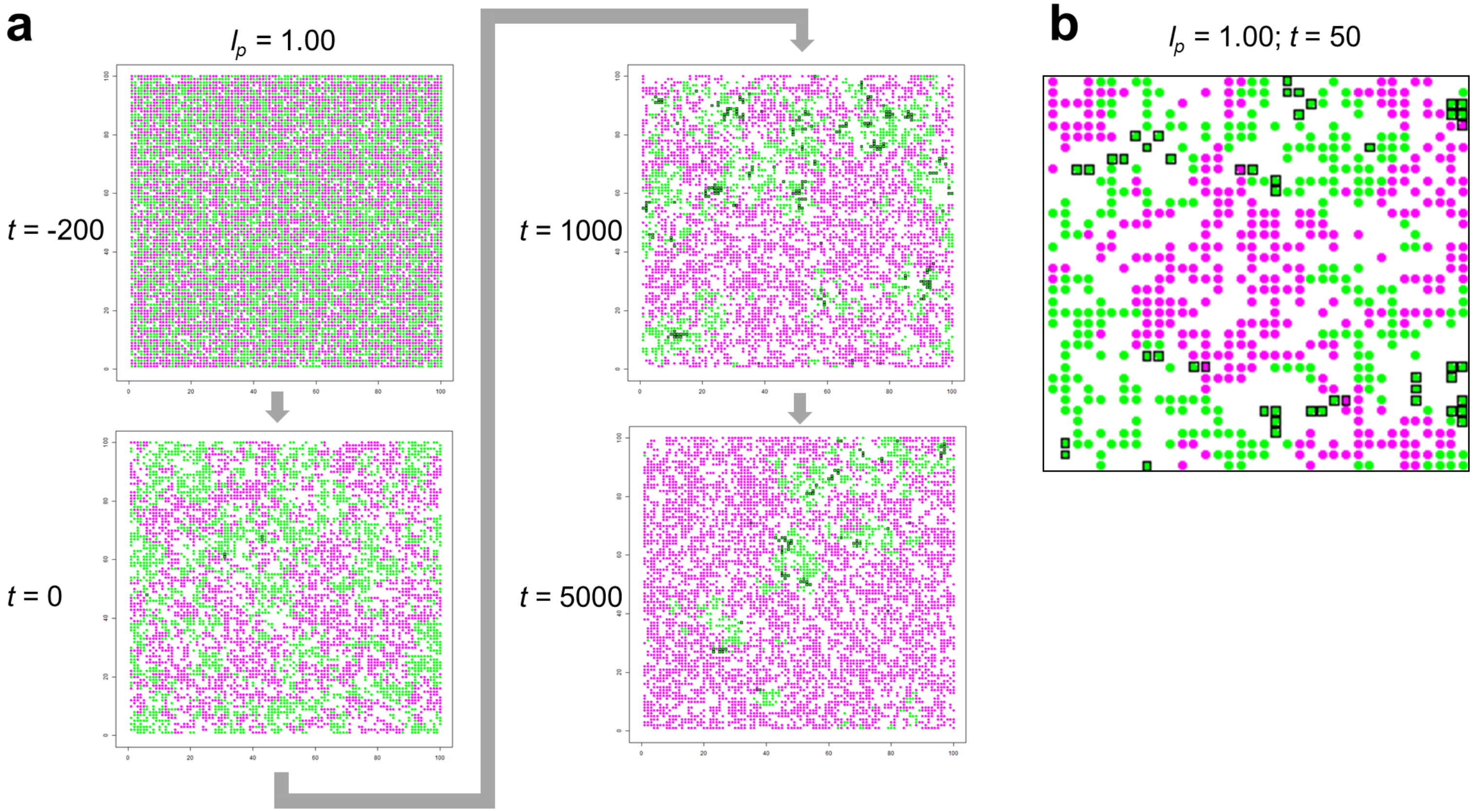

Supplementary Fig. 7. Magnified images from Fig. 6a (l_p = 1.00). **a** Time course of simulation results at l_p = 1.00. **b** Magnified image of a typical result at l_p = 1.00, shortly after the start of virus influx. Infected *SHR*(+) plants (magenta dots surrounded by black squares) appear only outside of the patches; plants inside the patches are not infected (magenta dots).

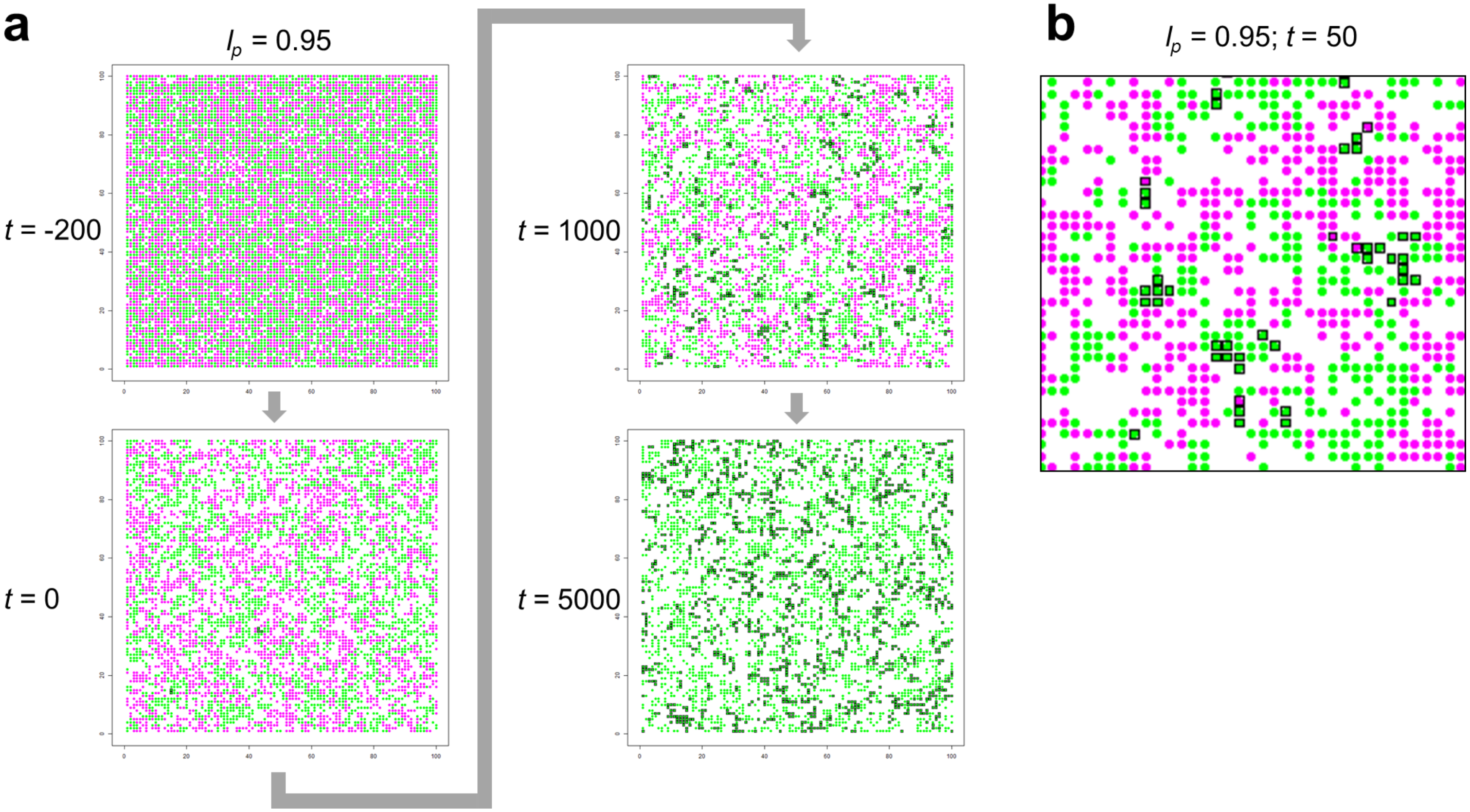

Supplementary Fig. 8. Magnified images from Fig. 6a (l_p = 0.95). a Time course of simulation results at l_p = 0.95. **b** Magnified image of a typical result at l_p = 0.95, shortly after the start of virus influx. Patches of *SHR*(+) plants (magenta) and *SHR*(–) plants (green) are smaller than those at l_p = 1.00.

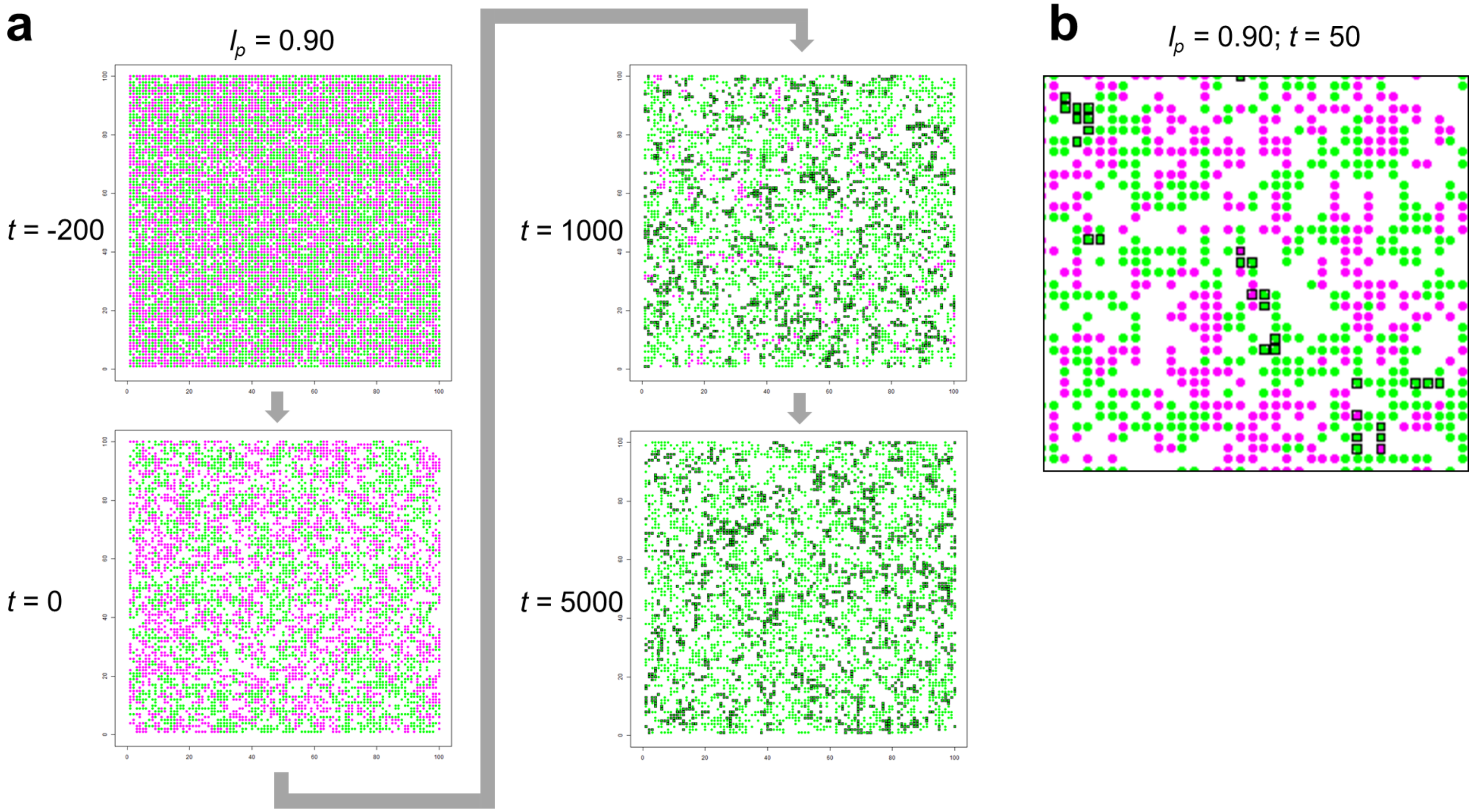

Supplementary Fig. 9. **Magnified images from Fig. 6a (l_p = 0.90). a** Time course of simulation results at l_p = 0.90. **b** Magnified image of a typical result at l_p = 0.90, shortly after the start of virus influx. Patches of *SHR*(+) plants (magenta) and *SHR*(–) plants (green) are smaller than those at l_p = 1.00 or 0.9

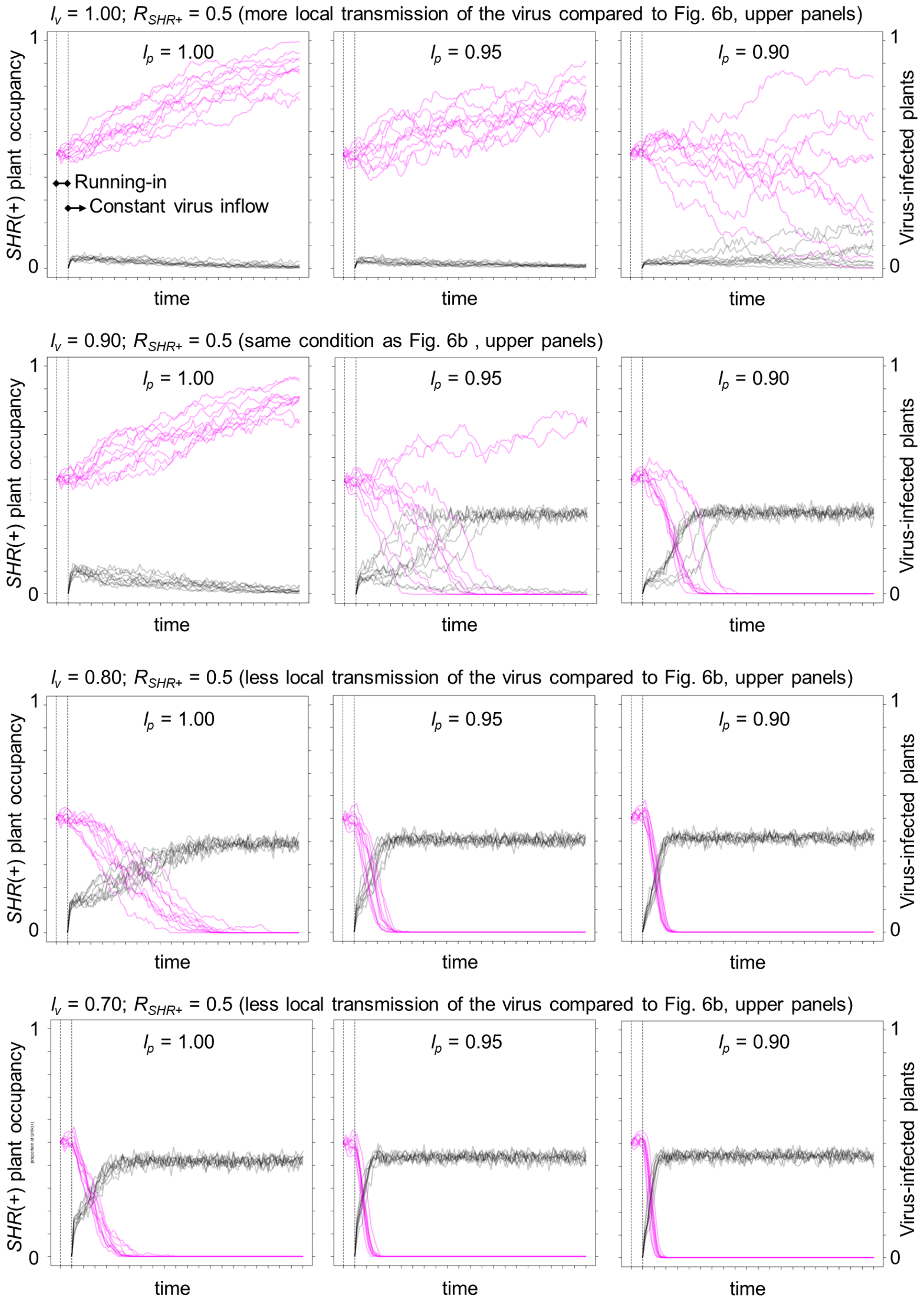

I_v = 1.00; R_{SHR+} = 0.5 (more local transmission of the virus compared to Fig. 6b, upper panels)
I_p = 1.00
I_p = 0.95
I_p = 0.90
Running-in
Constant virus inflow
SHR(+) plant occupancy
Virus-infected plants
1
0
time
I_v = 0.90; R_{SHR+} = 0.5 (same condition as Fig. 6b , upper panels)
I_p = 1.00
I_p = 0.95
I_p = 0.90
SHR(+) plant occupancy
Virus-infected plants
time
I_v = 0.80; R_{SHR+} = 0.5 (less local transmission of the virus compared to Fig. 6b, upper panels)
I_p = 1.00
I_p = 0.95
I_p = 0.90
SHR(+) plant occupancy
Virus-infected plants
time
I_v = 0.70; R_{SHR+} = 0.5 (less local transmission of the virus compared to Fig. 6b, upper panels)
I_p = 1.00
I_p = 0.95
I_p = 0.90
SHR(+) plant occupancy
Virus-infected plants
time

Supplementary Fig. 10. Simulations at different levels of dependency on local virus transmission (l_v) and local plant propagation (l_p). Summary of 10 simulation trials for each condition. Magenta lines indicate the occupancy of *SHR*(+) plants in comparison to the total. Black lines indicate the proportion of virus-infected plants among all plants.

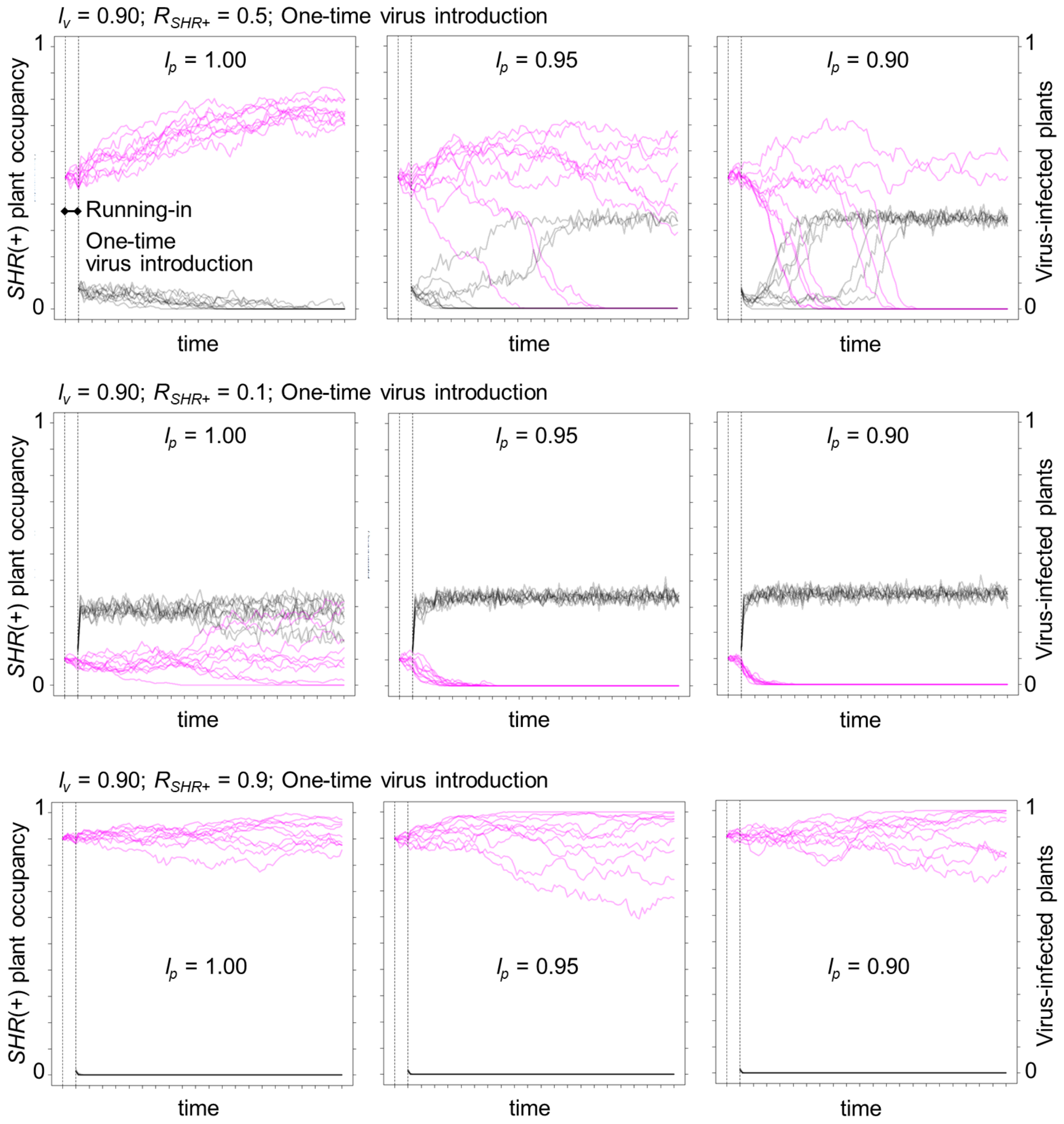

Supplementary Fig. 11. Simulations for one-time introduction of virus. Summary of 10 simulation trials for each condition. Magenta lines indicate the occupancy of *SHR*(+) plants in comparison to the total. Black lines indicate the proportion of virus-infected plants among all plants. Rapid extinction of the virus occurs when initial conditions of 90% *SHR*(+) plants (lower panels, R_{SHR+} = 0.9) were applied; the selection for *SHR*(+) plants does not function in these conditions, because of the lack of the virus.

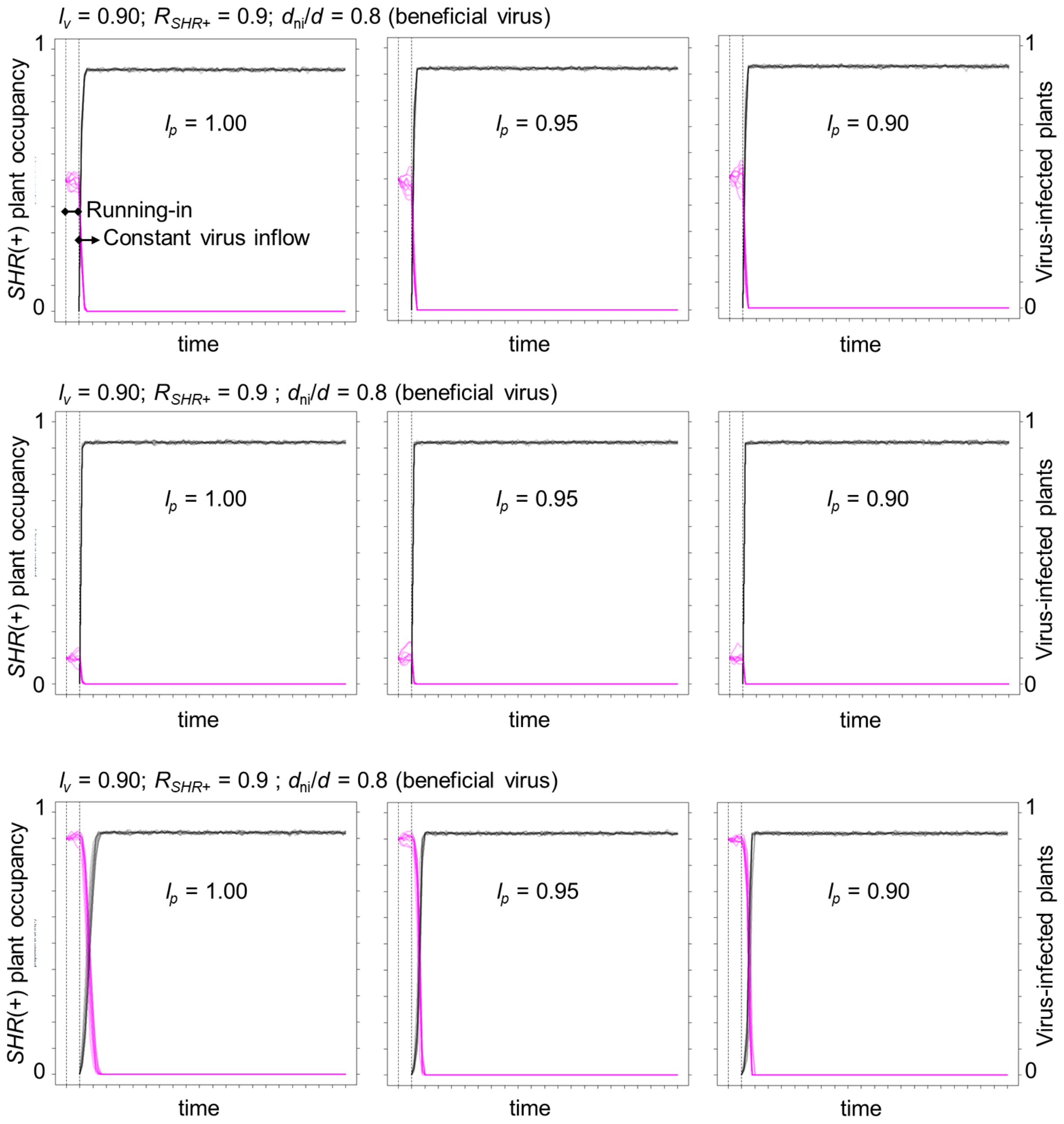

Supplementary Fig. 12. Simulations for beneficial virus infection. Summary of 10 simulation trials for each condition. Magenta lines indicate the occupancy of *SHR*(+) plants in comparison to the total. Black lines indicate the proportion of virus-infected plants among all plants.

Supplementary Table 1. Numbers of *N. benthamiana* (*R*+) plants showing a systemic hypersensitive response (SHR) after inoculation of wild-type (WT) and variant RNA3s, together with WT RNA1 and RNA2-YFP.

Days post inoculation	RNA3 Wild type	RNA3 CP-N31T	RNA3 CP-T45M
3	0/12 (0%)	0/12 (0%)	0/12 (0%)
4	0/12 (0%)	0/12 (0%)	11/12 (92%)
5	1/12 (8.3%)	0/12 (0%)	12/12 (100%)
6	2/12 (17%)	0/12 (0%)	12/12 (100%)
7	2/12 (17%)	0/12 (0%)	12/12 (100%)
8	2/12 (17%)	0/12 (0%)	12/12 (100%)

Supplementary Table 2. Number of infected sites and cells co-infected or exclusively infected by one of the RNA2 variants and estimated MOIs in *N. benthamiana* (*R*–) plants at 14–16 h after inoculation. YFP, yellow fluorescent protein; CFP, cyan fluorescent protein.

Inoculated RNA3	Sites or cells	Fluorescence type observed and total number of cells			
		YFP only	CFP only	YFP+CFP	Total
WT	Infected sites	6	1	63	70
	Estimated $\lambda_0 = 5.89 \pm 0.75$				
	Cell-0 + Cell-1+ Cell-2 (in 28 sites)	81	91	457	629
	Estimated $\lambda_{12} = 5.72 \pm 0.24$				
CP-N31T	Infected sites	4	2	27	33
	Estimated $\lambda_0 = 4.61 \pm 0.81$				
	Cell-0 + Cell-1+Cell-2 (in 10 sites)	28	50	197	273
	Estimated $\lambda_{12} = 5.75 \pm 0.36$				
CP-T45M	Infected sites	5	4	29	38
	Estimated $\lambda_0 = 4.01 \pm 0.66$				
	Cell-0 + Cell-1+Cell-2 (in 11 sites)	45	39	214	298
	Estimated $\lambda_{12} = 5.73 \pm 0.35$				

Supplementary Table 3. Number of infected sites and cells co-infected or exclusively infected by one of the RNA2 variants and estimated MOIs in *N. benthamiana* (*R*+) plants at 14–16 hours after inoculation.

Inoculated RNA3	Sites or cells	Fluorescence type observed and number of cells per class			
		YFP only	CFP only	YFP+CFP	Total
WT	Infected sites	12	15	40	67
	Estimated $\lambda_0 = 2.75 \pm 0.37$				
	Cell-0 + Cell-1+ Cell-2 (in 19 sites)	67	89	232	388
	Estimated $\lambda_{12} = 4.08 \pm 0.22$				
CP-N31T	Infected sites	7	9	45	61
	Estimated $\lambda_0 = 3.78 \pm 0.49$				
	Cell-0 + Cell-1+Cell-2 (in 18 sites)	60	65	322	447
	Estimated $\lambda_{12} = 5.67 \pm 0.28$				
CP-T45M	Infected sites	7	10	36	53
	Estimated $\lambda_0 = 3.31 \pm 0.26$				
	Cell-0 + Cell-1+Cell-2 (in 15 sites)	57	73	258	388
	Estimated $\lambda_{12} = 5.00 \pm 0.26$				

Supplementary Table 4. Population occupancy of *SHR*(+) plants at t = 5,000 units. Data are means ± standard deviation.

	Conditions			
l_p	1 (R_{SHR+} = 0.5)	2 (R_{SHR+} = 0.1)	3 (R_{SHR+} = 0.9)	4 (R_{SHR+} = 0.5; 5× virus influx)
l_p = 1.00	85.6 ± 6.2%	13.9 ± 16.3%	96.3 ± 4.9%	54.9 ± 28.5%
l_p = 0.95	15.1 ± 31.9%	0%	97.6 ± 3.2%	0%
l_p = 0.90	0%	0%	85.7 ± 17.9%	0%

Supplementary Table 5. Cumulative number of *SHR*(+) plants in the simulated time period under condition 2 (R_{SHR+} = 0.1).

l_p	Average cumulative number	Standard deviation
l_p = 1.00	2.54×10^{6} *	2.23×10^{6}
l_p = 0.95	1.22×10^{5}	0.42×10^{5}
l_p = 0.90	6.40×10^{4}	2.23×10^{4}

*Nine of 10 trials did not experience extinction; far more progeny would be reproduced before extinction

Supplementary Text 1

An R script for MOI estimation

```
## This script can be also found at Github (https://github.com/ShuheiMiyashita/Suicidal_Resistance2021) and at Zenodo
(https://doi.org/10.5281/zenodo.5105622).

### Data input
m0 <- 63; s0 <- 7; sites012 <- 28; m012 <- 457; s012 <- 172    #for N. benthamiana(R-) - wildtype RNA3
#NOTE: select one line for other combinations by removing first "#" in each line:
#m0 <- 27; s0 <- 6; sites012 <- 10; m012 <- 197; s012 <- 78    #for N. benthamiana(R-) - RNA3 CP-N31T
#m0 <- 29; s0 <- 9; sites012 <- 11; m012 <- 214; s012 <- 84    #for N. benthamiana(R-) - RNA3 CP-T45M
#m0 <- 40; s0 <- 27; sites012 <- 19; m012 <- 232; s012 <- 156  #for N. benthamiana(R+) - wildtype RNA3
#m0 <- 45; s0 <- 16; sites012 <- 18; m012 <- 322; s012 <- 125  #for N. benthamiana(R+) - RNA3 CP-N31T
#m0 <- 36; s0 <- 17; sites012 <- 15; m012 <- 258; s012 <- 130  #for N. benthamiana(R+) - RNA3 CP-T45M

# m0: no. of co-infected sites
# s0: no. of singly infected sites
# sites12: no. of co-infected sites with 10-30 infected cells at 14-16 hours after inoculation, used for lambda12
estimation
# m012: no. of co-infected cells
# s012: no. of singly infected cells
n012 <- m012+s012

### Main body of MOI estimation
n012 <- m012+s012
cell0 <- sites012               #number of cell-0 cells in sites012
cell1 <- sites012*8             #number of cell-1 cells in sites012
cell2 <- n012-sites012*9        #number of cell-2 cells in sites012

K <- 30 #maximum number of founders included in the calculation
kv <- NULL
lv <- NULL
for (i in 0:K){
kv <- c(kv,rep(i,i+1))
lv <- c(lv,0:i)
}
klv <- kv-lv
lklv <- lv*klv
ln <- (K+2)*(K+1)/2

## Function for log likelihood for any la,bda0 and lambda12
MOImLL <- function(lambda){
lambda0 <- lambda[1]; lambda1 <- lambda[2]; lambda2 <- lambda[2]; r0 <- 0.5
table0 <- matrix(rep(0,ln*5),ncol=5)
table0[,1] <- kv
table0[,2] <- lv
table0[,3] <- klv
table0[,4] <- lklv
table0[,5] <- dpois(table0[,1],lambda0)*dbinom(table0[,2],table0[,1],r0)
pni0 <- table0[1,5]
py0 <- sum(table0[which(table0[,2]>0&table0[,3]==0),5])/(1-pni0)
pc0 <- sum(table0[which(table0[,2]==0&table0[,3]>0),5])/(1-pni0)
pm0 <- 1-py0-pc0
ps0 <- py0+pc0
table0m <- table0[which(table0[,4]>0),]
table0m[,5] <- table0m[,5]/sum(table0m[,5])
table1 <- matrix(rep(0,ln*5),ncol=5)
table1[,1] <- kv
table1[,2] <- lv
table1[,3] <- klv
```

```
table1[,4] <- lklv
for (l in 1:nrow(table0m)){
table1[,5] <-
table1[,5]+table0m[l,5]*dpois(table1[,1],lambda1)*dbinom(table1[,2],table1[,1],table0m[l,2]/table0m[l,1])
}
pni1 <- table1[1,5]
py1 <- sum(table1[which(table1[,2]>0&table1[,3]==0),5])/(1-pni1)
pc1 <- sum(table1[which(table1[,2]==0&table1[,3]>0),5])/(1-pni1)
pm1 <- 1-py1-pc1
ps1 <- py1 + pc1
table1i <- table1[2:ln,]
table1i[,5] <- table1i[,5]/sum(table1i[,5])
table2 <- matrix(rep(0,ln*5),ncol=5)
table2[,1] <- kv
table2[,2] <- lv
table2[,3] <- klv
table2[,4] <- lklv
for (l in 1:(ln-1)){
table2[,5] <-
table2[,5]+table1i[l,5]*dpois(table2[,1],lambda2)*dbinom(table2[,2],table2[,1],table1i[l,2]/table1i[l,1])
}
pni2 <- table2[1,5]
py2 <- sum(table2[which(table2[,2]>0&table2[,3]==0),5])/(1-pni2)
pc2 <- sum(table2[which(table2[,2]==0&table2[,3]>0),5])/(1-pni2)
pm2 <- 1-py2-pc2
ps2 <- py2 + pc2

pm012 <- (cell0+cell1*pm1+cell2*pm2)/n012
ps012 <- (cell1*ps1+cell2*ps2)/n012
logL0 <- dmultinom(c(s0,m0),prob=c(ps0,pm0),log=TRUE)
logL012 <- dmultinom(c(s012,m012),prob=c(ps012,pm012),log=TRUE)
LL <- logL0 + logL012
-LL
}

## Maximization of log likelihood to find most likely lambda
init <- c(5,5)
M0lmLL.opt <- optim(init,M0lmLL, NULL, method="L-BFGS-B", hessian = TRUE, lower=c(0.1,0.1), upper=c(10,10))
M0lmLL.opt$par  # lambda0 and lambda12 estimates
v <- solve(M0lmLL.opt$hessian)
se <- sqrt(diag(v))
se              # lambda0 and lambda12 standard errors
```

Supplementary Text 2

An R script for simulation of suicidal population resistance of *SHR*(+) plants

```
## This script can be also found at Github (https://github.com/ShuheiMiyashita/Suicidal_Resistance2021) and at Zenodo (https://doi.org/10.5281/zenodo.5105622).

## CAUTION: Many files will be produced automatically. Before you start simulation, please (make and) select a directory to which the files will be saved. To complete the simulation below, several hours may be required, if you use a standard laptop. A modified R script for testing parameters manually without producing files (but showing Fig. 6-like results) can be found as Supplenentary Text 3.

#### initial settings
s <- 100      # size of lattice
rp <- 0.3     # reproduction rate of plant
rv <- 1.2     # reproduction rate of virus
lv <- 0.9     # dependency on local reproduction of virus
d <- 0.1      # mortality of plant without infection
dni <- 0.2    # mortality of SHR(-) plant upon viral infection
dpi <- 1      # mortality of SHR(+) plant upon viral infection
vi <- 0.001   # rate of virus influx


#### simulations in different conditions for Rshrp and lp
for (Rshrp in c(0.1,0.5,0.9)){ #Rshrp indicates initial proportion of SHR(+) plants
pt20rec <- NULL #for recording extinction time
ptcrec <- NULL #for recording cumulative number of SHR(-) and SHR(+) plants
for (lp in c(1,0.95,0.9)){ #lp indicates dependency on local reproduction of plants
for (seed in 1:10){ #10 trials
###initial settings for each trial
set.seed(seed)
rptv <- NULL # for recording plant and virus abundance at different time points
pt20rec <- c(pt20rec,lp,seed) #registration of current trial
pt20 <- 1 # 1: before extinction; 0: after extinction of SHR(+) plants
ptc <- c(0,0) #for recording cumulative number of SHR(-) and SHR(+) plants

### running-in without virus
tr <- 0 # time after starting running-in
pt <- matrix(rep(1,s*s),nrow=s)  #plant table; 0: open box, 1: SHR(-), and 2: SHR(+)
pt[sample(1:(s*s),round(s*s*Rshrp,0),replace=F)] <- 2  #introducing SHR(+) plants according to lp
while (tr < 201){
pt <- pt*rbinom(s*s,1,1-d) # death without virus
## visualization
if (tr%%50==0){
pname <- paste("plot",seed,"_",Rshrp*100,"_",lp*100,"_tr",tr,".png",sep="")
png(pname,width=800,height=800)
plot(0,0,xlim=c(0,s),ylim=c(0,s),type="n",xlab="",ylab="")
for (x in 1:s){
points(rep(x,s),1:s,col=rgb((1-pt[x,])^2,1-pt[x,]*(pt[x,]-1)/2,(1-pt[x,])^2),pch=19,cex=1) # SHR(-): green; SHR(+): magenta
}
dev.off()
ptv <- c(tr,length(which(pt==0)),length(which(pt==1)),length(which(pt==2)),0)
rptv <- rbind(rptv,ptv)
}else{
}
## plant propagation
pto <- (pt-1)*(pt-2)/2                                # finding open box
plt <- pt[c(s,1:(s-1)),]*rbinom(s*s,1,rp*lp/4)*pto    # local propagation from top box
plb <- pt[c(2:s,1),]*rbinom(s*s,1,rp*lp/4)*pto        # local propagation from bottom box
pll <- pt[,c(s,1:(s-1))]*rbinom(s*s,1,rp*lp/4)*pto    # local propagation from left box
plr <- pt[,c(2:s,1)]*rbinom(s*s,1,rp*lp/4)*pto        # local propagation from right box
```

```
pgp <- sample(1:(s*s),rbinom(1,sum((pt-1)*pt/2),rp*(1-lp)),replace=F); pgpv <- rep(0,s*s); pgpv[pgp] <- 2 # global propagation of SHR(+) plant
pgn <- sample(1:(s*s),rbinom(1,sum((pt-2)^2*pt),rp*(1-lp)),replace=F); pgnv <- rep(0,s*s); pgnv[pgn] <- 1 # global propagation of SHR(-) plant
## deciding which parent leave progeny
pv <- rbind(as.vector(plt),as.vector(plb),as.vector(pll),as.vector(plr),pgpv*as.vector(pto),pgnv*as.vector(pto))
cspv <- colSums(pv)
pvp <-  1-(pv-1)*(pv-2)/2                       # returns 1 if a box has plant inhabitant
cspvp <- colSums(pvp)                           # number of inhabited boxes
prv <- rep(0,s*s)                               # vector for propagation result
prv[which(cspvp==1)] <- cspv[which(cspvp==1)]  # boxes with only one parent candidate
for (i in which(cspvp > 1)){                    # boxes with multiple parent candidates
prv[i] <- sample(pv[,i],1,prob=pvp[,i])        # random decision of parents
}
pt <- pt+prv
tr <- tr+1
}

### main part after starting virus introduction
t <- 0 # time after starting virus introduction
vt <- matrix(rep(0,s*s),nrow=s) # generating virus table and virus introduction; 0: no infection and 1: infection

while (t < 5001){
##constant virus influx
cvi <- rbinom(s*s,1,vi)*(1-vt)*(1-(pt-1)*(pt-2)/2) #0.1% of plants are randomly challenged by the virus
vt <- vt+cvi
## death
drt <- ((1-vt)*d+vt*pt*(pt-1)^2*dpi/2+vt*pt*(pt-2)^2*dni)*ceiling(pt/2) # mortality table
pt <- pt*rbinom(s*s,1,1-drt)
## viral propagation
pti <- 1-(pt-1)*(pt-2)/2                                    # boxes inhabited
vt <- vt*pti                                                # updating vt by removing dead plant
vlt <- vt[c(s,1:(s-1)),]*rbinom(s*s,1,rv*lv/4)              # local spread from top box
vlb <- vt[c(2:s,1),]*rbinom(s*s,1,rv*lv/4)                  # local spread from bottom box
vll <- vt[,c(s,1:(s-1))]*rbinom(s*s,1,rv*lv/4)              # local spread from left box
vlr <- vt[,c(2:s,1)]*rbinom(s*s,1,rv*lv/4)                  # local spread from right box
vg <- sample(1:(s*s),rbinom(1,sum(vt),rv*(1-lv)),replace=F)  # global spread
vgv <- rep(0,s*s); vgv[vg] <- 1                             # global spread vector
vv <- as.vector(vlt)+as.vector(vlb)+as.vector(vll)+as.vector(vlr)+vgv # sum
vt <- vt+vv                                                 # addition allowing >1
vt <- ceiling(vt/6)*pti     # updating vt by limiting to plant-inhabiting boxes, without allowing >1
## plots and record
if (t%%50==0){
pname <- paste("plot",seed,"_",Rshrp*100,"_",lp*100,"_t",t,".png",sep="")
png(pname,width=800,height=800)
plot(0,0,xlim=c(0,s),ylim=c(0,s),type="n",xlab="",ylab="")
for (x in 1:s){
points(rep(x,s),1:s,col=rgb((1-pt[x,])^2,1-pt[x,]*(pt[x,]-1)/2,(1-pt[x,])^2),pch=19,cex=1) # SHR(-): green; SHR(+): magenta
points(rep(x,s),1:s,col=rgb(0,0,0,alpha=vt[x,]),pch=22,cex=1)
}
dev.off()
ptv <- c(200+t,length(which(pt==0)),length(which(pt==1)),length(which(pt==2)),sum(vt))
rptv <- rbind(rptv,ptv)
}else{
}
## plant propagation
pto <- (pt-1)*(pt-2)/2 # finding open box
plt <- pt[c(s,1:(s-1)),]*rbinom(s*s,1,rp*lp/4)*pto       # local propagation from top box
plb <- pt[c(2:s,1),]*rbinom(s*s,1,rp*lp/4)*pto           # local propagation from bottom box
pll <- pt[,c(s,1:(s-1))]*rbinom(s*s,1,rp*lp/4)*pto       # local propagation from left box
plr <- pt[,c(2:s,1)]*rbinom(s*s,1,rp*lp/4)*pto           # local propagation from right box
pgp <- sample(1:(s*s),rbinom(1,sum((pt-1)*pt/2),rp*(1-lp)),replace=F); pgpv <- rep(0,s*s); pgpv[pgp] <- 2 # global propagation of SHR(+) plant
```

```
pgn <- sample(1:(s*s),rbinom(1,sum((pt-2)^2*pt),rp*(1-lp)),replace=F); pgnv <- rep(0,s*s); pgnv[pgn] <- 1 # global
propagation of SHR(-) plant
## deciding which parent leave progeny
pv <- rbind(as.vector(plt),as.vector(plb),as.vector(pll),as.vector(plr),pgpv*as.vector(pto),pgnv*as.vector(pto))
cspv <- colSums(pv)
pvp <-  1-(pv-1)*(pv-2)/2                       # returns 1 if a box has plant inhabitant
cspvp <- colSums(pvp)                           # number of inhabited boxes
prv <- rep(0,s*s)                               # vector for propagation result
prv[which(cspvp==1)] <- cspv[which(cspvp==1)]  # boxes with only one parent candidate
for (i in which(cspvp > 1)){                    # boxes with multiple parent candidates
prv[i] <- sample(pv[,i],1,prob=pvp[,i])        # random decision of parent
}
pt <- pt+prv
ptc <- ptc+c(length(which(pt==1)),length(which(pt==2))) # updating cumulative number
## detecting and recording extinction
if(length(which(pt==2))==0&&pt20==1){
pt20rec <- c(pt20rec,t)
pt20 <- 0
}else{
}
t <- t+1
}
ptcrec <- rbind(ptcrec,ptc) #recording cumulative numbers

### outputting the record of plant and virus abundance for each trial
fname <- paste("output",seed,"_",Rshrp*100,"_",lp*100,".csv",sep="")
write.csv(rptv,file=fname,row.names=F)
}
}

### outputting the record of extinction time
cname1 <- paste("pt20rec_",Rshrp*100,".csv",sep="")
write.csv(pt20rec,file=cname1,row.names=F)

### outputting the record of cumulative number
cname2 <- paste("ptcrec_",Rshrp*100,".csv",sep="")
write.csv(ptcrec,file=cname2,row.names=F)
}


#### summary of outputs
###vidualization
for (k in c(10,50,90)){
for (j in c(90,95,100)){
pname <- paste("fig",k,"_",j,".png",sep="")
png(pname,width=800,height=800)
plot(0,0,xlim=c(0,105),ylim=c(0,1),type="n",xlab="time",ylab="proportion of SHR(+)",xaxt="n",yaxt="n")
axis(side=1,at=0:21*5,labels=F)
axis(side=2,at=0:10*0.1,labels=F)
axis(side=4,at=0:10*0.1,labels=F)
for (i in 1:10){
fname <- paste("output",i,"_",k,"_",j,".csv",sep="")
tt <- read.csv(fname)
par(new=T)
plot(0:105,tt[1:106,4]/(tt[1:106,3]+tt[1:106,4]),pch=19,xlim=c(0,105),ylim=c(0,1),col=rgb(1,0,1,alpha=0.3),type="l",l
wd=3,ann=F,xlab="",ylab="",xaxt="n",yaxt="n")
par(new=T)
plot(5:105,tt[6:106,5]/(tt[6:106,3]+tt[6:106,4]),pch=19,xlim=c(0,105),ylim=c(0,1),col=rgb(0,0,0,alpha=0.2),type="l",l
wd=3,ann=F,xlab="",ylab="",xaxt="n",yaxt="n")
}
abline(v=5,lty=2)
abline(v=0,lty=2)
dev.off()
}
```

```
}

### occupancy
tvr <- NULL
for (k in c(10,50,90)){
for (j in c(90,95,100)){
tv <- NULL
for (i in 1:10){
fname <- paste("output",i,"_",k,"_",j,".csv",sep="")
tt <- read.csv(fname)
tv <- c(tv,tt[106,4]/(tt[106,3]+tt[106,4]))
}
tvr <- rbind(tvr,c(mean(tv),sd(tv)))
}
}
write.csv(tvr,file="final_occupancy.csv")
```

Supplementary Text 3

A modified R script for simulation of suicidal population resistance of *SHR*(+) plants, without producing files

```
## This script can be also found at Github (https://github.com/ShuheiMiyashita/Suicidal_Resistance2021) and at Zenodo (https://doi.org/10.5281/zenodo.5105622).

## This script is for testing different parameters manually. For making multiple trials with different lp or Rshrp values, please refer Supplementary Text 2.

#### initial settings
s <- 100      # size of lattice
Rshrp <- 0.5  # initial proportion of SHR(+) plants
rp <- 0.3     # reproduction rate of plant
rv <- 1.2     # reproduction rate of virus
lp <- 1.0     # dependency on local reproduction of plants
lv <- 0.9     # dependency on local reproduction of virus
d <- 0.1      # mortality of plant without infection
dni <- 0.2    # mortality of SHR(-) plant upon viral infection
dpi <- 1      # mortality of SHR(+) plant upon viral infection
vi <- 0.001   # rate of virus influx


#### main body of simulation
###initial settings for each trial
rptv <- NULL # for recording plant and virus abundance at different time points

### running-in without virus
tr <- 0 # time after starting running-in
pt <- matrix(rep(1,s*s),nrow=s)  #plant table; 0: open box, 1: SHR(-), and 2: SHR(+)
pt[sample(1:(s*s),round(s*s*Rshrp,0),replace=F)] <- 2  #introducing SHR(+) plants according to lp
while (tr < 201){
pt <- pt*rbinom(s*s,1,1-d) # death without virus
## visualization
if (tr%%50==0){
plot(0,0,xlim=c(0,s),ylim=c(0,s),type="n",xlab="",ylab="")
for (x in 1:s){
points(rep(x,s),1:s,col=rgb((1-pt[x,])^2,1-pt[x,]*(pt[x,]-1)/2,(1-pt[x,])^2),pch=19,cex=0.5) # SHR(-): green; SHR(+): magenta
}
ptv <- c(tr,length(which(pt==0)),length(which(pt==1)),length(which(pt==2)),0)
rptv <- rbind(rptv,ptv)
}else{
}
## plant propagation
pto <- (pt-1)*(pt-2)/2                                 # finding open box
plt <- pt[c(s,1:(s-1)),]*rbinom(s*s,1,rp*lp/4)*pto      # local propagation from top box
plb <- pt[c(2:s,1),]*rbinom(s*s,1,rp*lp/4)*pto          # local propagation from bottom box
pll <- pt[,c(s,1:(s-1))]*rbinom(s*s,1,rp*lp/4)*pto      # local propagation from left box
plr <- pt[,c(2:s,1)]*rbinom(s*s,1,rp*lp/4)*pto          # local propagation from right box
pgp <- sample(1:(s*s),rbinom(1,sum((pt-1)*pt/2),rp*(1-lp)),replace=F); pgpv <- rep(0,s*s); pgpv[pgp] <- 2 # global propagation of SHR(+) plant
pgn <- sample(1:(s*s),rbinom(1,sum((pt-2)^2*pt),rp*(1-lp)),replace=F); pgnv <- rep(0,s*s); pgnv[pgn] <- 1 # global propagation of SHR(-) plant
## deciding which parent leave progeny
pv <- rbind(as.vector(plt),as.vector(plb),as.vector(pll),as.vector(plr),pgpv*as.vector(pto),pgnv*as.vector(pto))
cspv <- colSums(pv)
pvp <-  1-(pv-1)*(pv-2)/2                   # returns 1 if a box has plant inhabitant
cspvp <- colSums(pvp)                       # number of inhabited boxes
prv <- rep(0,s*s)                           # vector for propagation result
```

```
prv[which(cspvp==1)] <- cspv[which(cspvp==1)]  # boxes with only one parent candidate
for (i in which(cspvp > 1)){                   # boxes with multiple parent candidates
prv[i] <- sample(pv[,i],1,prob=pvp[,i])        # random decision of parents
}
pt <- pt+prv
tr <- tr+1
}

### main part after starting virus introduction
t <- 0 # time after starting virus introduction
vt <- matrix(rep(0,s*s),nrow=s) # generating virus table and virus introduction; 0: no infection and 1: infection

while (t < 5001){
##constant virus influx
cvi <- rbinom(s*s,1,vi)*(1-vt)*(1-(pt-1)*(pt-2)/2) #0.1% of plants are randomly challenged by the virus
vt <- vt+cvi
## death
drt <- ((1-vt)*d+vt*pt*(pt-1)^2*dpi/2+vt*pt*(pt-2)^2*dni)*ceiling(pt/2) # mortality table
pt <- pt*rbinom(s*s,1,1-drt)
## viral propagation
pti <- 1-(pt-1)*(pt-2)/2                                    # boxes inhabited
vt <- vt*pti                                                # updating vt by removing dead plant
vlt <- vt[c(s,1:(s-1)),]*rbinom(s*s,1,rv*lv/4)              # local spread from top box
vlb <- vt[c(2:s,1),]*rbinom(s*s,1,rv*lv/4)                  # local spread from bottom box
vll <- vt[,c(s,1:(s-1))]*rbinom(s*s,1,rv*lv/4)              # local spread from left box
vlr <- vt[,c(2:s,1)]*rbinom(s*s,1,rv*lv/4)                  # local spread from right box
vg <- sample(1:(s*s),rbinom(1,sum(vt),rv*(1-lv)),replace=F)  # global spread
vgv <- rep(0,s*s); vgv[vg] <- 1                             # global spread vector
vv <- as.vector(vlt)+as.vector(vlb)+as.vector(vll)+as.vector(vlr)+vgv # sum
vt <- vt+vv                                                 # addition allowing >1
vt <- ceiling(vt/6)*pti     # updating vt by limiting to plant-inhabiting boxes, without allowing >1
## plots and record
if (t%%50==0){
plot(0,0,xlim=c(0,s),ylim=c(0,s),type="n",xlab="",ylab="")
for (x in 1:s){
points(rep(x,s),1:s,col=rgb((1-pt[x,])^2,1-pt[x,]*(pt[x,]-1)/2,(1-pt[x,])^2),pch=19,cex=0.5) # SHR(-): green; SHR(+): magenta
points(rep(x,s),1:s,col=rgb(0,0,0,alpha=vt[x,]),pch=22,cex=0.5)
}
ptv <- c(200+t,length(which(pt==0)),length(which(pt==1)),length(which(pt==2)),sum(vt))
rptv <- rbind(rptv,ptv)
}else{
}
## plant propagation
pto <- (pt-1)*(pt-2)/2 # finding open box
plt <- pt[c(s,1:(s-1)),]*rbinom(s*s,1,rp*lp/4)*pto       # local propagation from top box
plb <- pt[c(2:s,1),]*rbinom(s*s,1,rp*lp/4)*pto           # local propagation from bottom box
pll <- pt[,c(s,1:(s-1))]*rbinom(s*s,1,rp*lp/4)*pto       # local propagation from left box
plr <- pt[,c(2:s,1)]*rbinom(s*s,1,rp*lp/4)*pto           # local propagation from right box
pgp <- sample(1:(s*s),rbinom(1,sum((pt-1)*pt/2),rp*(1-lp)),replace=F); pgpv <- rep(0,s*s); pgpv[pgp] <- 2 # global propagation of SHR(+) plant
pgn <- sample(1:(s*s),rbinom(1,sum((pt-2)^2*pt),rp*(1-lp)),replace=F); pgnv <- rep(0,s*s); pgnv[pgn] <- 1 # global propagation of SHR(-) plant
## deciding which parent leave progeny
pv <- rbind(as.vector(plt),as.vector(plb),as.vector(pll),as.vector(plr),pgpv*as.vector(pto),pgnv*as.vector(pto))
cspv <- colSums(pv)
pvp <-  1-(pv-1)*(pv-2)/2                      # returns 1 if a box has plant inhabitant
cspvp <- colSums(pvp)                          # number of inhabited boxes
prv <- rep(0,s*s)                              # vector for propagation result
prv[which(cspvp==1)] <- cspv[which(cspvp==1)]  # boxes with only one parent candidate
for (i in which(cspvp > 1)){                   # boxes with multiple parent candidates
prv[i] <- sample(pv[,i],1,prob=pvp[,i])        # random decision of parent
}
pt <- pt+prv
```

```
t <- t+1
}
```

Supplementary Text 4

An R script for simulation of suicidal population resistance of *SHR*(+) plants with one-time virus introduction

```
## This script can be also found at Github (https://github.com/ShuheiMiyashita/Suicidal_Resistance2021) and at Zenodo
(https://doi.org/10.5281/zenodo.5105622).

## CAUTION: Many files will be produced automatically. Before you start simulation, please (make and) select a
directory to which the files will be saved. To complete the simulation below, several hours may be required, if you
use a standard laptop. A modified R script for testing parameters manually without producing files (but showing Fig.
6-like results) can be found as Supplementary Text 3.

#### initial settings
s <- 100       # size of lattice
rp <- 0.3      # reproduction rate of plant
rv <- 1.2      # reproduction rate of virus
lv <- 0.9      # dependency on local reproduction of virus
d <- 0.1       # mortality of plant without infection
dni <- 0.2     # mortality of SHR(-) plant upon viral infection
dpi <- 1       # mortality of SHR(+) plant upon viral infection
vo <- 0.1      # one-time virus introduction


#### simulations in different conditions for Rshrp and lp
for (Rshrp in c(0.1,0.5,0.9)){ #Rshrp indicates initial proportion of SHR(+) plants
pt20rec <- NULL #for recording extinction time
ptcrec <- NULL #for recording cumulative number of SHR(-) and SHR(+) plants
for (lp in c(1,0.95,0.9)){ #lp indicates dependency on local reproduction of plants
for (seed in 1:10){ #10 trials
###initial settings for each trial
set.seed(seed)
rptv <- NULL # for recording plant and virus abundance at different time points
pt20rec <- c(pt20rec,lp,seed) #registration of current trial
pt20 <- 1 # 1: before extinction; 0: after extinction of SHR(+) plants
ptc <- c(0,0) #for recording cumulative number of SHR(-) and SHR(+) plants

### running-in without virus
tr <- 0 # time after starting running-in
pt <- matrix(rep(1,s*s),nrow=s)  #plant table; 0: open box, 1: SHR(-), and 2: SHR(+)
pt[sample(1:(s*s),round(s*s*Rshrp,0),replace=F)] <- 2  #introducing SHR(+) plants according to lp
while (tr < 201){
pt <- pt*rbinom(s*s,1,1-d) # death without virus
## visualization
if (tr%%50==0){
pname <- paste("plot_o",seed,"_",Rshrp*100,"_",lp*100,"_tr",tr,".png",sep="")
png(pname,width=800,height=800)
plot(0,0,xlim=c(0,s),ylim=c(0,s),type="n",xlab="",ylab="")
for (x in 1:s){
points(rep(x,s),1:s,col=rgb((1-pt[x,])^2,1-pt[x,]*(pt[x,]-1)/2,(1-pt[x,])^2),pch=19,cex=1) # SHR(-): green; SHR(+):
magenta
}
dev.off()
ptv <- c(tr,length(which(pt==0)),length(which(pt==1)),length(which(pt==2)),0)
rptv <- rbind(rptv,ptv)
}else{
}
## plant propagation
pto <- (pt-1)*(pt-2)/2                              # finding open box
plt <- pt[c(s,1:(s-1)),]*rbinom(s*s,1,rp*lp/4)*pto   # local propagation from top box
plb <- pt[c(2:s,1),]*rbinom(s*s,1,rp*lp/4)*pto       # local propagation from bottom box
```

```
pll <- pt[,c(s,1:(s-1))]*rbinom(s*s,1,rp*lp/4)*pto       # local propagation from left box
plr <- pt[,c(2:s,1)]*rbinom(s*s,1,rp*lp/4)*pto           # local propagation from right box
pgp <- sample(1:(s*s),rbinom(1,sum((pt-1)*pt/2),rp*(1-lp)),replace=F); pgpv <- rep(0,s*s); pgpv[pgp] <- 2 # global propagation of SHR(+) plant
pgn <- sample(1:(s*s),rbinom(1,sum((pt-2)^2*pt),rp*(1-lp)),replace=F); pgnv <- rep(0,s*s); pgnv[pgn] <- 1 # global propagation of SHR(-) plant
## deciding which parent leave progeny
pv <- rbind(as.vector(plt),as.vector(plb),as.vector(pll),as.vector(plr),pgpv*as.vector(pto),pgnv*as.vector(pto))
cspv <- colSums(pv)
pvp <-  1-(pv-1)*(pv-2)/2                        # returns 1 if a box has plant inhabitant
cspvp <- colSums(pvp)                            # number of inhabited boxes
prv <- rep(0,s*s)                                # vector for propagation result
prv[which(cspvp==1)] <- cspv[which(cspvp==1)]  # boxes with only one parent candidate
for (i in which(cspvp > 1)){                     # boxes with multiple parent candidates
prv[i] <- sample(pv[,i],1,prob=pvp[,i])          # random decision of parents
}
pt <- pt+prv
tr <- tr+1
}

### main part after starting virus introduction
t <- 0 # time after starting virus introduction
vt <- matrix(rep(0,s*s),nrow=s) # generating virus table and virus introduction; 0: no infection and 1: infection
tvo <- rbinom(s*s,1,vo)*(1-vt)*(1-(pt-1)*(pt-2)/2) #10% of plants are randomly challenged by the virus
vt <- vt+tvo


while (t < 5001){
## death
drt <- ((1-vt)*d+vt*pt*(pt-1)^2*dpi/2+vt*pt*(pt-2)^2*dni)*ceiling(pt/2) # mortality table
pt <- pt*rbinom(s*s,1,1-drt)
## viral propagation
pti <- 1-(pt-1)*(pt-2)/2                                      # boxes inhabited
vt <- vt*pti                                                  # updating vt by removing dead plant
vlt <- vt[c(s,1:(s-1)),]*rbinom(s*s,1,rv*lv/4)                # local spread from top box
vlb <- vt[c(2:s,1),]*rbinom(s*s,1,rv*lv/4)                    # local spread from bottom box
vll <- vt[,c(s,1:(s-1))]*rbinom(s*s,1,rv*lv/4)                # local spread from left box
vlr <- vt[,c(2:s,1)]*rbinom(s*s,1,rv*lv/4)                    # local spread from right box
vg <- sample(1:(s*s),rbinom(1,sum(vt),rv*(1-lv)),replace=F)  # global spread
vgv <- rep(0,s*s); vgv[vg] <- 1                               # global spread vector
vv <- as.vector(vlt)+as.vector(vlb)+as.vector(vll)+as.vector(vlr)+vgv # sum
vt <- vt+vv                                                   # addition allowing >1
vt <- ceiling(vt/6)*pti     # updating vt by limiting to plant-inhabiting boxes, without allowing >1
## plots and record
if (t%%50==0){
pname <- paste("plot_o",seed,"_",Rshrp*100,"_",lp*100,"_t",t,".png",sep="")
png(pname,width=800,height=800)
plot(0,0,xlim=c(0,s),ylim=c(0,s),type="n",xlab="",ylab="")
for (x in 1:s){
points(rep(x,s),1:s,col=rgb((1-pt[x,])^2,1-pt[x,]*(pt[x,]-1)/2,(1-pt[x,])^2),pch=19,cex=1) # SHR(-): green; SHR(+): magenta
points(rep(x,s),1:s,col=rgb(0,0,0,alpha=vt[x,]),pch=22,cex=1)
}
dev.off()
ptv <- c(200+t,length(which(pt==0)),length(which(pt==1)),length(which(pt==2)),sum(vt))
rptv <- rbind(rptv,ptv)
}else{
}
## plant propagation
pto <- (pt-1)*(pt-2)/2 # finding open box
plt <- pt[c(s,1:(s-1)),]*rbinom(s*s,1,rp*lp/4)*pto       # local propagation from top box
plb <- pt[c(2:s,1),]*rbinom(s*s,1,rp*lp/4)*pto           # local propagation from bottom box
pll <- pt[,c(s,1:(s-1))]*rbinom(s*s,1,rp*lp/4)*pto       # local propagation from left box
plr <- pt[,c(2:s,1)]*rbinom(s*s,1,rp*lp/4)*pto           # local propagation from right box
```

```
pgp <- sample(1:(s*s),rbinom(1,sum((pt-1)*pt/2),rp*(1-lp)),replace=F); pgpv <- rep(0,s*s); pgpv[pgp] <- 2 # global propagation of SHR(+) plant
pgn <- sample(1:(s*s),rbinom(1,sum((pt-2)^2*pt),rp*(1-lp)),replace=F); pgnv <- rep(0,s*s); pgnv[pgn] <- 1 # global propagation of SHR(-) plant
## deciding which parent leave progeny
pv <- rbind(as.vector(plt),as.vector(plb),as.vector(pll),as.vector(plr),pgpv*as.vector(pto),pgnv*as.vector(pto))
cspv <- colSums(pv)
pvp <-  1-(pv-1)*(pv-2)/2                      # returns 1 if a box has plant inhabitant
cspvp <- colSums(pvp)                          # number of inhabited boxes
prv <- rep(0,s*s)                              # vector for propagation result
prv[which(cspvp==1)] <- cspv[which(cspvp==1)]  # boxes with only one parent candidate
for (i in which(cspvp > 1)){                   # boxes with multiple parent candidates
prv[i] <- sample(pv[,i],1,prob=pvp[,i])        # random decision of parent
}
pt <- pt+prv
ptc <- ptc+c(length(which(pt==1)),length(which(pt==2))) # updating cumulative number
## detecting and recording extinction
if(length(which(pt==2))==0&&pt20==1){
pt20rec <- c(pt20rec,t)
pt20 <- 0
}else{
}
t <- t+1
}
ptcrec <- rbind(ptcrec,ptc) #recording cumulative numbers

### outputting the record of plant and virus abundance for each trial
fname <- paste("output_o",seed,"_",Rshrp*100,"_",lp*100,".csv",sep="")
write.csv(rptv,file=fname,row.names=F)
}
}

### outputting the record of extinction time
cname1 <- paste("pt20rec_o_",Rshrp*100,".csv",sep="")
write.csv(pt20rec,file=cname1,row.names=F)

### outputting the record of cumulative number
cname2 <- paste("ptcrec_o_",Rshrp*100,".csv",sep="")
write.csv(ptcrec,file=cname2,row.names=F)
}


#### summary of outputs
###vidualization
for (k in c(10,50,90)){
for (j in c(90,95,100)){
pname <- paste("fig_o",k,"_",j,".png",sep="")
png(pname,width=800,height=800)
plot(0,0,xlim=c(0,105),ylim=c(0,1),type="n",xlab="time",ylab="proportion of SHR(+)",xaxt="n",yaxt="n")
axis(side=1,at=0:21*5,labels=F)
axis(side=2,at=0:10*0.1,labels=F)
axis(side=4,at=0:10*0.1,labels=F)
for (i in 1:10){
fname <- paste("output_o",i,"_",k,"_",j,".csv",sep="")
tt <- read.csv(fname)
par(new=T)
plot(0:105,tt[1:106,4]/(tt[1:106,3]+tt[1:106,4]),pch=19,xlim=c(0,105),ylim=c(0,1),col=rgb(1,0,1,alpha=0.3),type="l",lwd=3,ann=F,xlab="",ylab="",xaxt="n",yaxt="n")
par(new=T)
plot(5:105,tt[6:106,5]/(tt[6:106,3]+tt[6:106,4]),pch=19,xlim=c(0,105),ylim=c(0,1),col=rgb(0,0,0,alpha=0.2),type="l",lwd=3,ann=F,xlab="",ylab="",xaxt="n",yaxt="n")
}
abline(v=5,lty=2)
abline(v=0,lty=2)
```

```
dev.off()
}
}

### occupancy
tvr <- NULL
for (k in c(10,50,90)){
for (j in c(90,95,100)){
tv <- NULL
for (i in 1:10){
fname <- paste("output_o",i,"_",k,"_",j,".csv",sep="")
tt <- read.csv(fname)
tv <- c(tv,tt[106,4]/(tt[106,3]+tt[106,4]))
}
tvr <- rbind(tvr,c(mean(tv),sd(tv)))
}
}
write.csv(tvr,file="final_occupancy_o.csv")
```

Supplementary Text 5

An R script for simulation of suicidal population resistance of *SHR*(+) plants with a beneficial virus

```
## This script can be also found at Github (https://github.com/ShuheiMiyashita/Suicidal_Resistance2021) and at Zenodo
(https://doi.org/10.5281/zenodo.5105622).

## CAUTION: Many files will be produced automatically. Before you start simulation, please (make and) select a
directory to which the files will be saved. To complete the simulation below, several hours may be required, if you
use a standard laptop. A modified R script for testing parameters manually without producing files (but showing Fig.
6-like results) can be found as Supplementary Text 3.

#### initial settings
s <- 100      # size of lattice
rp <- 0.3     # reproduction rate of plant
rv <- 1.2     # reproduction rate of virus
lv <- 0.9     # dependency on local reproduction of virus
d <- 0.1      # mortality of plant without infection
dni <- 0.08    # mortality of SHR(-) plant upon viral infection
dpi <- 1      # mortality of SHR(+) plant upon viral infection
vi <- 0.001   # rate of virus influx


#### simulations in different conditions for Rshrp and lp
for (Rshrp in c(0.1,0.5,0.9)){ #Rshrp indicates initial proportion of SHR(+) plants
pt20rec <- NULL #for recording extinction time
ptcrec <- NULL #for recording cumulative number of SHR(-) and SHR(+) plants
for (lp in c(1,0.95,0.9)){ #lp indicates dependency on local reproduction of plants
for (seed in 1:10){ #10 trials
###initial settings for each trial
set.seed(seed)
rptv <- NULL # for recording plant and virus abundance at different time points
pt20rec <- c(pt20rec,lp,seed) #registration of current trial
pt20 <- 1 # 1: before extinction; 0: after extinction of SHR(+) plants
ptc <- c(0,0) #for recording cumulative number of SHR(-) and SHR(+) plants

### running-in without virus
tr <- 0 # time after starting running-in
pt <- matrix(rep(1,s*s),nrow=s)  #plant table; 0: open box, 1: SHR(-), and 2: SHR(+)
pt[sample(1:(s*s),round(s*s*Rshrp,0),replace=F)] <- 2  #introducing SHR(+) plants according to lp
while (tr < 201){
pt <- pt*rbinom(s*s,1,1-d) # death without virus
## visualization
if (tr%%50==0){
pname <- paste("plot_b",seed,"_",Rshrp*100,"_",lp*100,"_tr",tr,".png",sep="")
png(pname,width=800,height=800)
plot(0,0,xlim=c(0,s),ylim=c(0,s),type="n",xlab="",ylab="")
for (x in 1:s){
points(rep(x,s),1:s,col=rgb((1-pt[x,])^2,1-pt[x,]*(pt[x,]-1)/2,(1-pt[x,])^2),pch=19,cex=1) # SHR(-): green; SHR(+):
magenta
}
dev.off()
ptv <- c(tr,length(which(pt==0)),length(which(pt==1)),length(which(pt==2)),0)
rptv <- rbind(rptv,ptv)
}else{
}
## plant propagation
pto <- (pt-1)*(pt-2)/2                          # finding open box
plt <- pt[c(s,1:(s-1)),]*rbinom(s*s,1,rp*lp/4)*pto      # local propagation from top box
plb <- pt[c(2:s,1),]*rbinom(s*s,1,rp*lp/4)*pto          # local propagation from bottom box
```

```
pll <- pt[,c(s,1:(s-1))]*rbinom(s*s,1,rp*lp/4)*pto       # local propagation from left box
plr <- pt[,c(2:s,1)]*rbinom(s*s,1,rp*lp/4)*pto           # local propagation from right box
pgp <- sample(1:(s*s),rbinom(1,sum((pt-1)*pt/2),rp*(1-lp)),replace=F); pgpv <- rep(0,s*s); pgpv[pgp] <- 2 # global
propagation of SHR(+) plant
pgn <- sample(1:(s*s),rbinom(1,sum((pt-2)^2*pt),rp*(1-lp)),replace=F); pgnv <- rep(0,s*s); pgnv[pgn] <- 1 # global
propagation of SHR(-) plant
## deciding which parent leave progeny
pv <- rbind(as.vector(plt),as.vector(plb),as.vector(pll),as.vector(plr),pgpv*as.vector(pto),pgnv*as.vector(pto))
cspv <- colSums(pv)
pvp <-  1-(pv-1)*(pv-2)/2                        # returns 1 if a box has plant inhabitant
cspvp <- colSums(pvp)                            # number of inhabited boxes
prv <- rep(0,s*s)                                # vector for propagation result
prv[which(cspvp==1)] <- cspv[which(cspvp==1)]  # boxes with only one parent candidate
for (i in which(cspvp > 1)){                     # boxes with multiple parent candidates
prv[i] <- sample(pv[,i],1,prob=pvp[,i])          # random decision of parents
}
pt <- pt+prv
tr <- tr+1
}

### main part after starting virus introduction
t <- 0 # time after starting virus introduction
vt <- matrix(rep(0,s*s),nrow=s) # generating virus table and virus introduction; 0: no infection and 1: infection

while (t < 5001){
##constant virus influx
cvi <- rbinom(s*s,1,vi)*(1-vt)*(1-(pt-1)*(pt-2)/2) #0.1% of plants are randomly attacked by the virus
vt <- vt+cvi
## death
drt <- ((1-vt)*d+vt*pt*(pt-1)^2*dpi/2+vt*pt*(pt-2)^2*dni)*ceiling(pt/2) # mortality table
pt <- pt*rbinom(s*s,1,1-drt)
## viral propagation
pti <- 1-(pt-1)*(pt-2)/2                                       # boxes inhabited
vt <- vt*pti                                                   # updating vt by removing dead plant
vlt <- vt[c(s,1:(s-1)),]*rbinom(s*s,1,rv*lv/4)                 # local spread from top box
vlb <- vt[c(2:s,1),]*rbinom(s*s,1,rv*lv/4)                     # local spread from bottom box
vll <- vt[,c(s,1:(s-1))]*rbinom(s*s,1,rv*lv/4)                 # local spread from left box
vlr <- vt[,c(2:s,1)]*rbinom(s*s,1,rv*lv/4)                     # local spread from right box
vg <- sample(1:(s*s),rbinom(1,sum(vt),rv*(1-lv)),replace=F)  # global spread
vgv <- rep(0,s*s); vgv[vg] <- 1                                # global spread vector
vv <- as.vector(vlt)+as.vector(vlb)+as.vector(vll)+as.vector(vlr)+vgv # sum
vt <- vt+vv                                                    # addition allowing >1
vt <- ceiling(vt/6)*pti     # updating vt by limiting to plant-inhabiting boxes, without allowing >1
## plots and record
if (t%%50==0){
pname <- paste("plot_b",seed,"_",Rshrp*100,"_",lp*100,"_t",t,".png",sep="")
png(pname,width=800,height=800)
plot(0,0,xlim=c(0,s),ylim=c(0,s),type="n",xlab="",ylab="")
for (x in 1:s){
points(rep(x,s),1:s,col=rgb((1-pt[x,])^2,1-pt[x,]*(pt[x,]-1)/2,(1-pt[x,])^2),pch=19,cex=1) # SHR(-): green; SHR(+):
magenta
points(rep(x,s),1:s,col=rgb(0,0,0,alpha=vt[x,]),pch=22,cex=1)
}
dev.off()
ptv <- c(200+t,length(which(pt==0)),length(which(pt==1)),length(which(pt==2)),sum(vt))
rptv <- rbind(rptv,ptv)
}else{
}
## plant propagation
pto <- (pt-1)*(pt-2)/2 # finding open box
plt <- pt[c(s,1:(s-1)),]*rbinom(s*s,1,rp*lp/4)*pto       # local propagation from top box
plb <- pt[c(2:s,1),]*rbinom(s*s,1,rp*lp/4)*pto           # local propagation from bottom box
pll <- pt[,c(s,1:(s-1))]*rbinom(s*s,1,rp*lp/4)*pto       # local propagation from left box
plr <- pt[,c(2:s,1)]*rbinom(s*s,1,rp*lp/4)*pto           # local propagation from right box
```

```
pgp <- sample(1:(s*s),rbinom(1,sum((pt-1)*pt/2),rp*(1-lp)),replace=F); pgpv <- rep(0,s*s); pgpv[pgp] <- 2 # global propagation of SHR(+) plant
pgn <- sample(1:(s*s),rbinom(1,sum((pt-2)^2*pt),rp*(1-lp)),replace=F); pgnv <- rep(0,s*s); pgnv[pgn] <- 1 # global propagation of SHR(-) plant
## deciding which parent leave progeny
pv <- rbind(as.vector(plt),as.vector(plb),as.vector(pll),as.vector(plr),pgpv*as.vector(pto),pgnv*as.vector(pto))
cspv <- colSums(pv)
pvp <-  1-(pv-1)*(pv-2)/2                       # returns 1 if a box has plant inhabitant
cspvp <- colSums(pvp)                           # number of inhabited boxes
prv <- rep(0,s*s)                               # vector for propagation result
prv[which(cspvp==1)] <- cspv[which(cspvp==1)]  # boxes with only one parent candidate
for (i in which(cspvp > 1)){                    # boxes with multiple parent candidates
prv[i] <- sample(pv[,i],1,prob=pvp[,i])        # random decision of parent
}
pt <- pt+prv
ptc <- ptc+c(length(which(pt==1)),length(which(pt==2))) # updating cumulative number
## detecting and recording extinction
if(length(which(pt==2))==0&&pt20==1){
pt20rec <- c(pt20rec,t)
pt20 <- 0
}else{
}
t <- t+1
}
ptcrec <- rbind(ptcrec,ptc) #recording cumulative numbers

### outputting the record of plant and virus abundance for each trial
fname <- paste("output_b",seed,"_",Rshrp*100,"_",lp*100,".csv",sep="")
write.csv(rptv,file=fname,row.names=F)
}
}

### outputting the record of extinction time
cname1 <- paste("pt20rec_b_",Rshrp*100,".csv",sep="")
write.csv(pt20rec,file=cname1,row.names=F)

### outputting the record of cumulative number
cname2 <- paste("ptcrec_b_",Rshrp*100,".csv",sep="")
write.csv(ptcrec,file=cname2,row.names=F)
}


#### summary of outputs
###vidualization
for (k in c(10,50,90)){
for (j in c(90,95,100)){
pname <- paste("fig_b",k,"_",j,".png",sep="")
png(pname,width=800,height=800)
plot(0,0,xlim=c(0,105),ylim=c(0,1),type="n",xlab="time",ylab="proportion of SHR(+)",xaxt="n",yaxt="n")
axis(side=1,at=0:21*5,labels=F)
axis(side=2,at=0:10*0.1,labels=F)
axis(side=4,at=0:10*0.1,labels=F)
for (i in 1:10){
fname <- paste("output_b",i,"_",k,"_",j,".csv",sep="")
tt <- read.csv(fname)
par(new=T)
plot(0:105,tt[1:106,4]/(tt[1:106,3]+tt[1:106,4]),pch=19,xlim=c(0,105),ylim=c(0,1),col=rgb(1,0,1,alpha=0.3),type="l",lwd=3,ann=F,xlab="",ylab="",xaxt="n",yaxt="n")
par(new=T)
plot(5:105,tt[6:106,5]/(tt[6:106,3]+tt[6:106,4]),pch=19,xlim=c(0,105),ylim=c(0,1),col=rgb(0,0,0,alpha=0.2),type="l",lwd=3,ann=F,xlab="",ylab="",xaxt="n",yaxt="n")
}
abline(v=5,lty=2)
abline(v=0,lty=2)
```

```
dev.off()
}
}

### occupancy
tvr <- NULL
for (k in c(10,50,90)){
for (j in c(90,95,100)){
tv <- NULL
for (i in 1:10){
fname <- paste("output_b",i,"_",k,"_",j,".csv",sep="")
tt <- read.csv(fname)
tv <- c(tv,tt[106,4]/(tt[106,3]+tt[106,4]))
}
tvr <- rbind(tvr,c(mean(tv),sd(tv)))
}
}

write.csv(tvr,file="final_occupancy_b.csv")
```

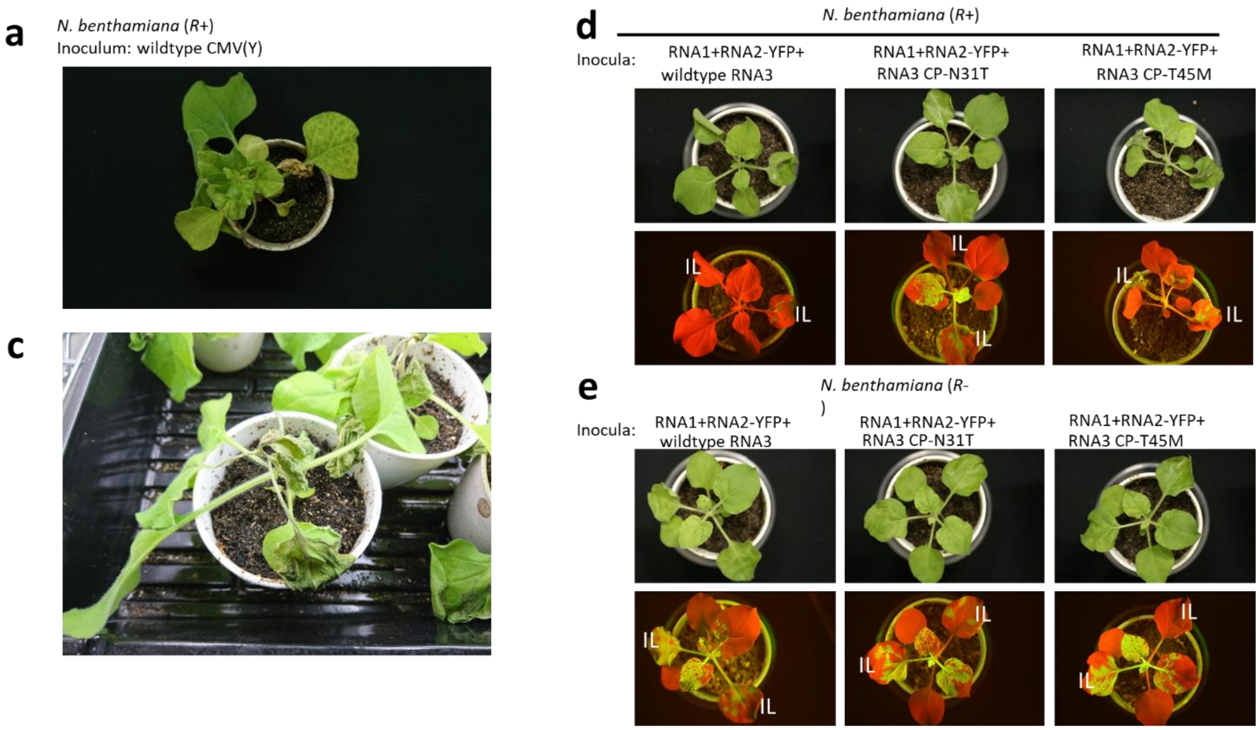

Supplementary Fig. 13. Uncropped, unedited images for Fig. 2.

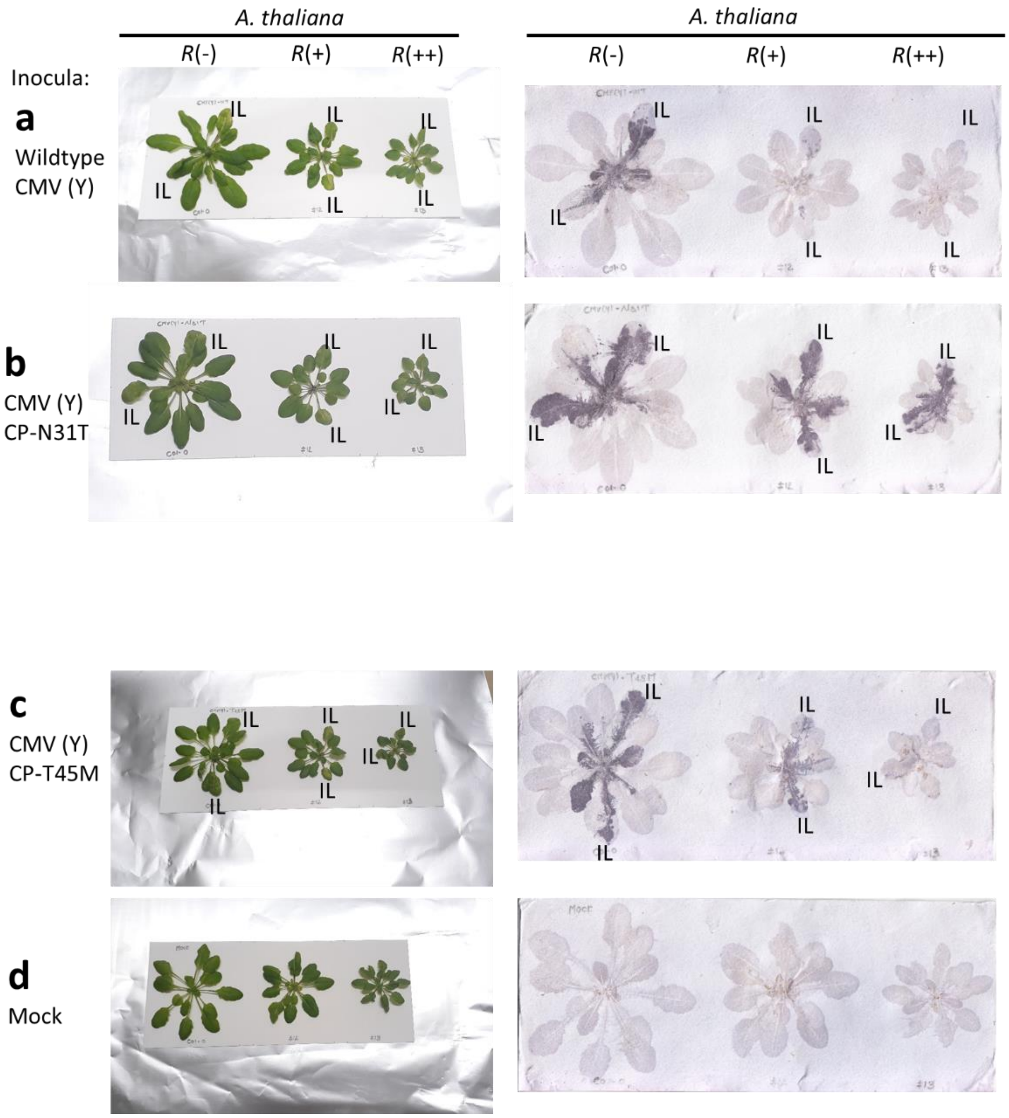

Supplementary Fig. 14. Uncropped, unedited images for Fig. 3.

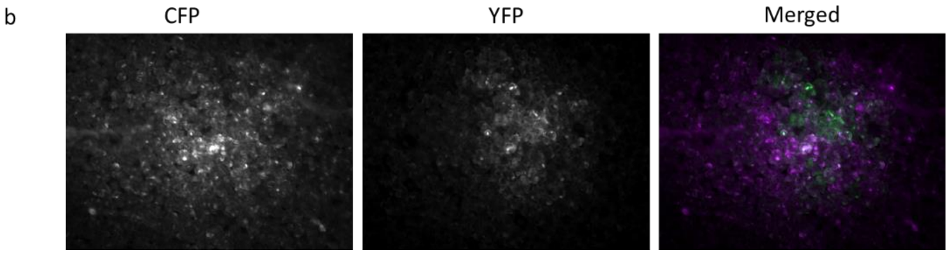

Supplementary Fig. 15. Uncropped, unedited images for Fig. 4.

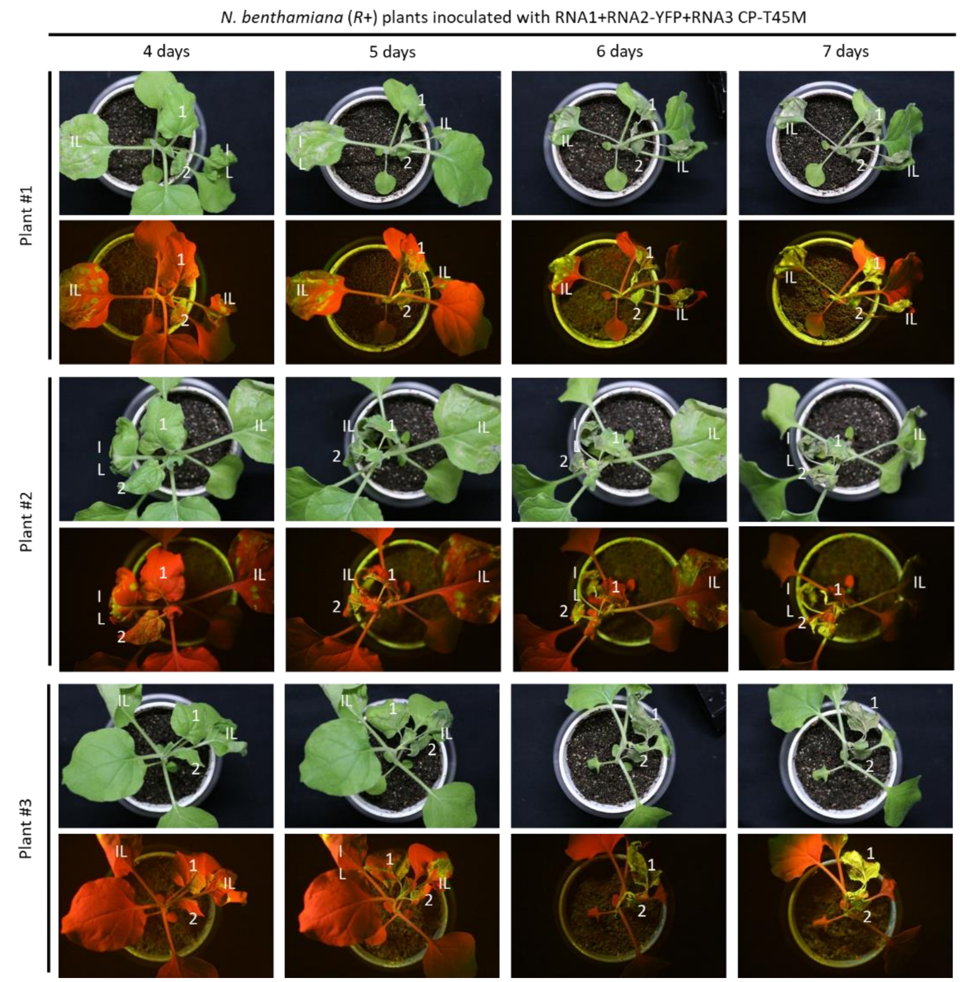

Supplementary Fig. 16. Uncropped, unedited images for Supplementary Fig. 1.

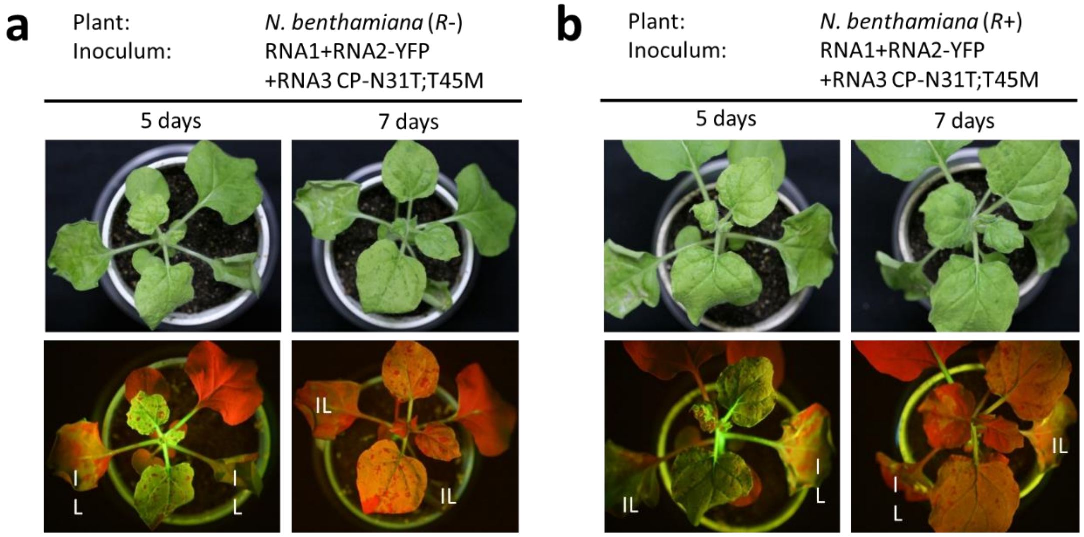

Supplementary Fig. 17. Uncropped, unedited images for Supplementary Fig. 2.

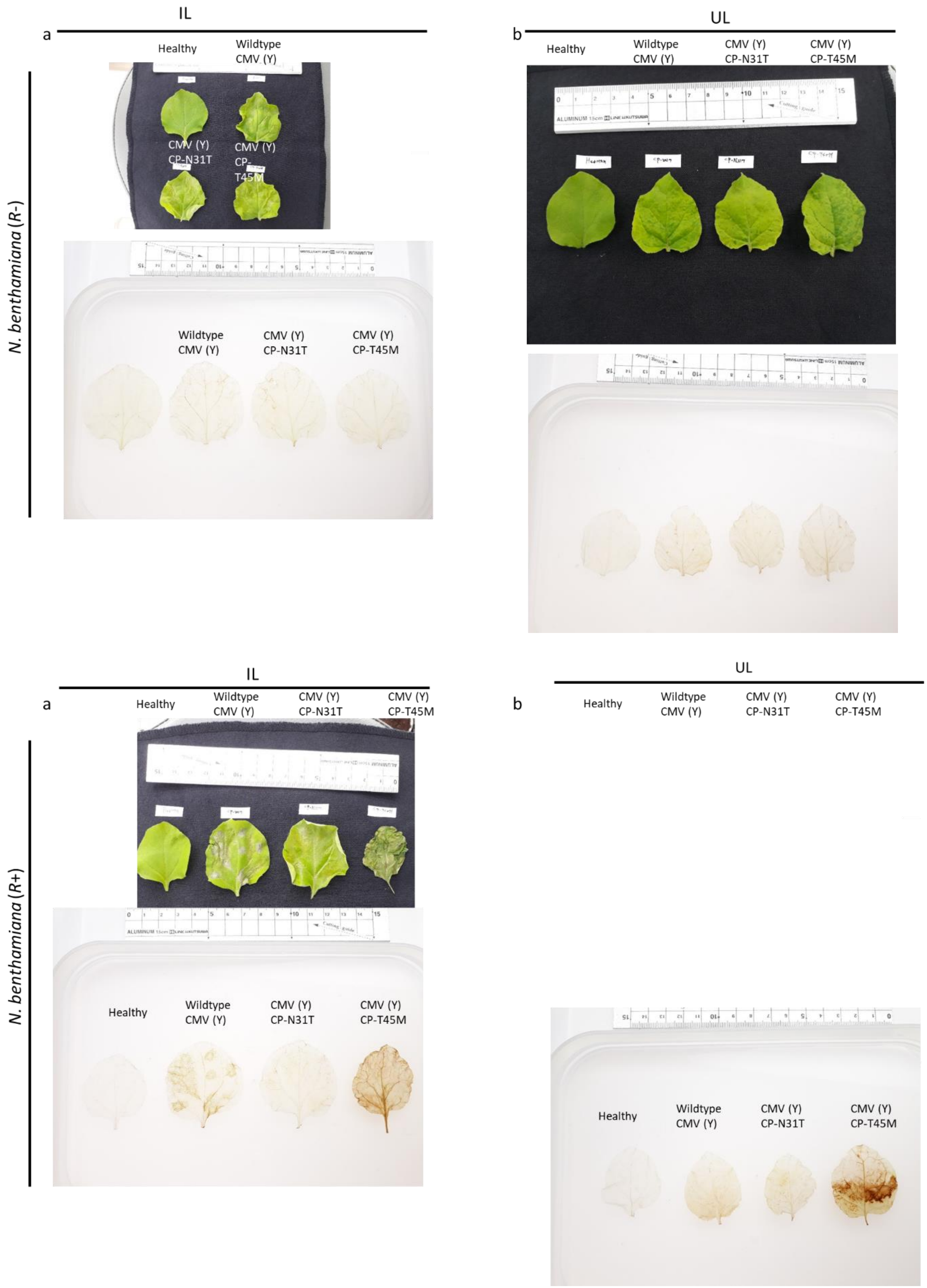

Supplementary Fig. 18. Uncropped, unedited images for Supplementary Fig. 3ab.

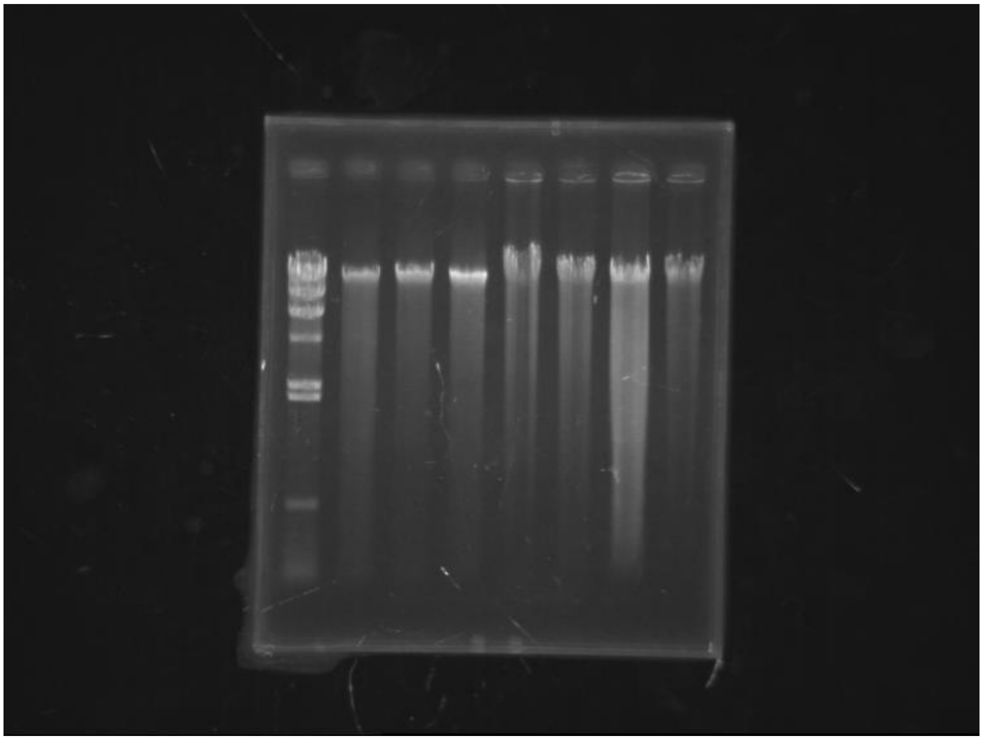

Supplementary Fig. 19. Uncropped, unedited image for Supplementary Fig. 3c.

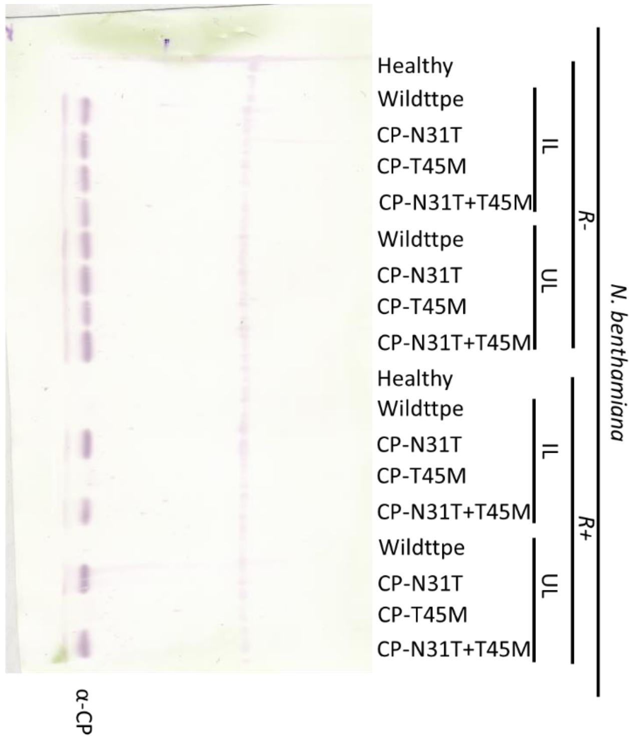

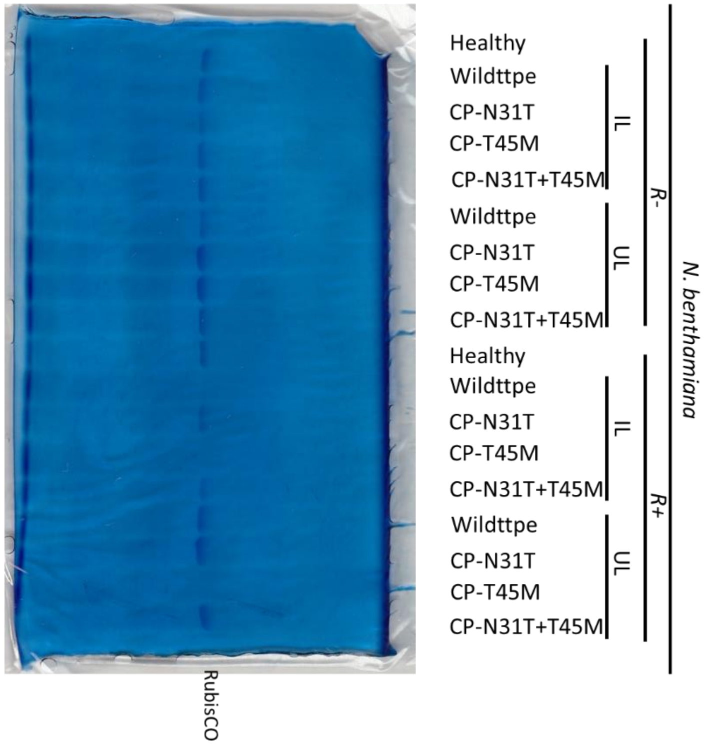

Supplementary Fig. 20. Uncropped, unedited images for Supplementary Fig. 4a.

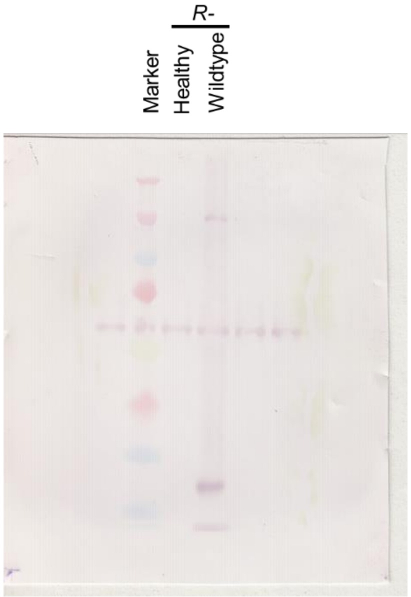

Supplementary Fig. 21. Uncropped, unedited image for Supplementary Fig. 4b.

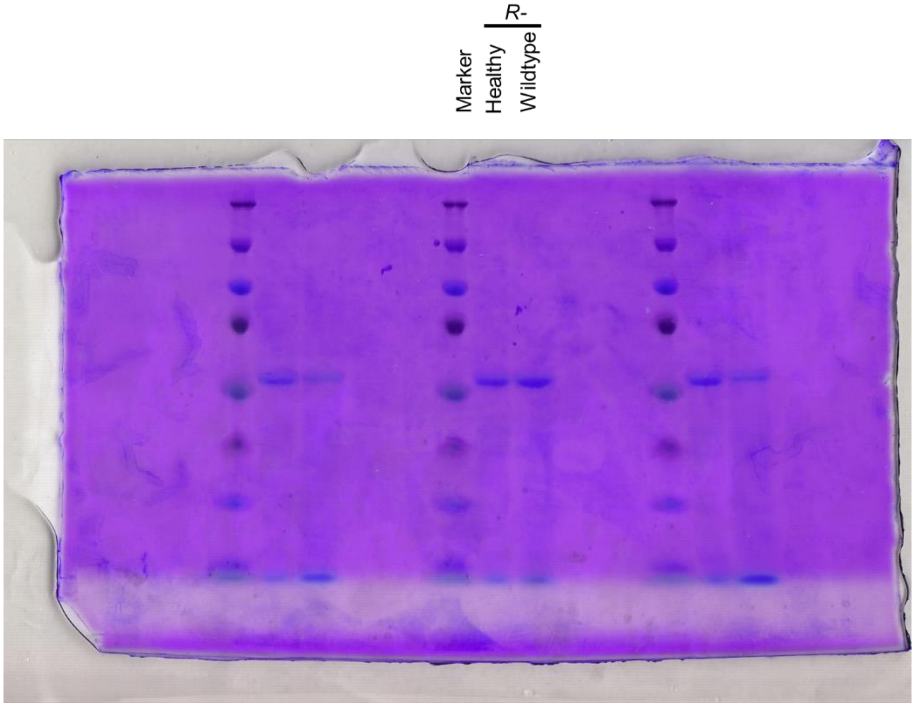

Supplementary Fig. 22. Uncropped, unedited image for Supplementary Fig. 4c.

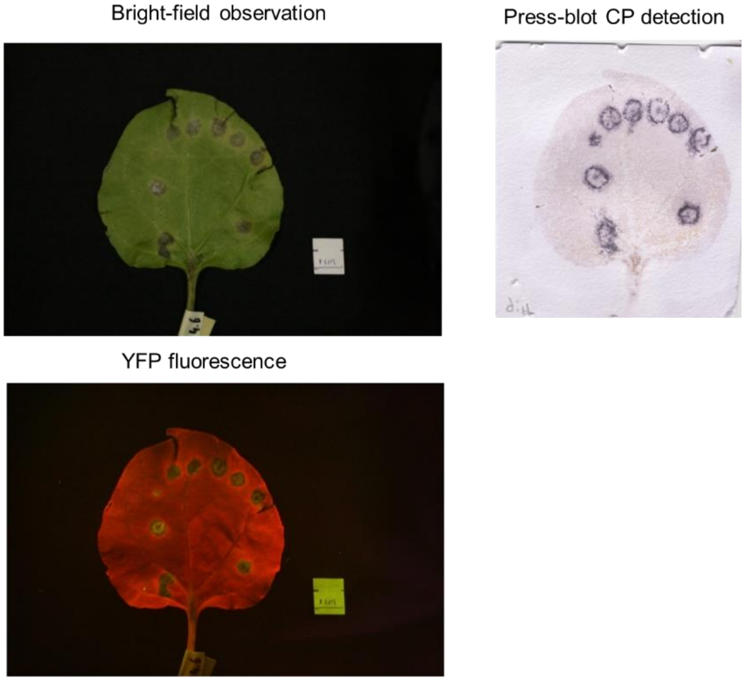

Supplementary Fig. 23. Uncropped, unedited images for Supplementary Fig. 4d.

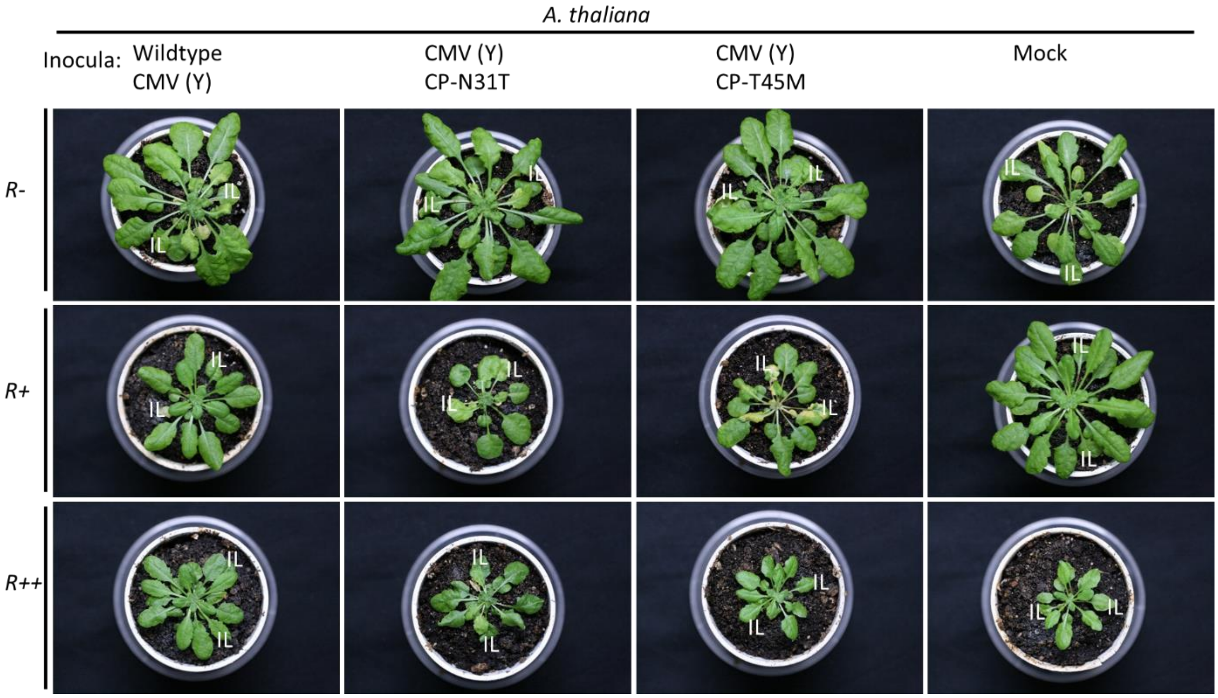

Supplementary Fig. 24. Uncropped, unedited images for Supplementary Fig. 5.

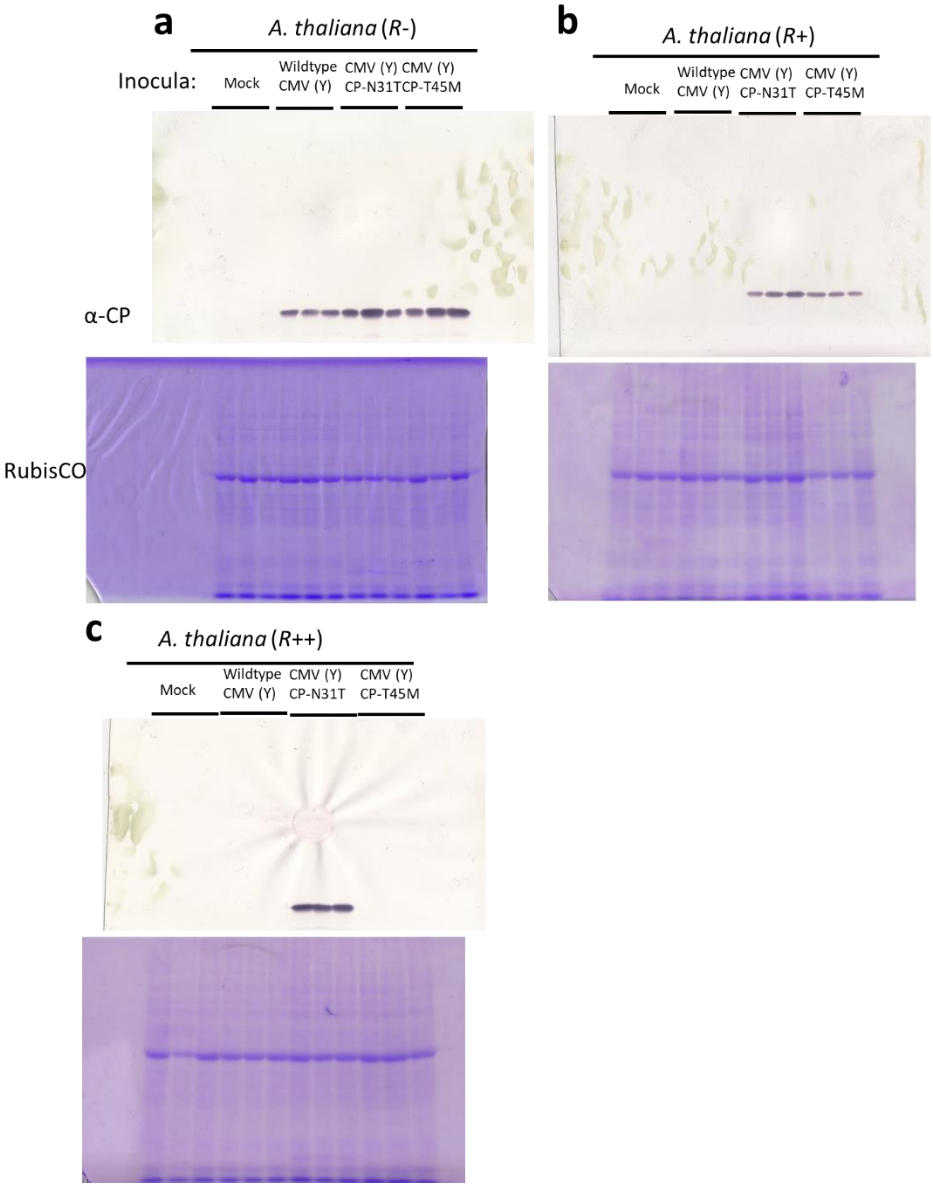

Supplementary Fig. 25. Uncropped, unedited images for Supplementary Fig. 6.
