# Supplementary material for: Plant death caused by inefficient induction of antiviral R-gene-mediated resistance may function as a suicidal population resistance mechanism
Source: Commun Biol. 2021 Aug 9;4:947. doi: 10.1038/s42003-021-02482-7 (PMC8352862; doi:10.1038/s42003-021-02482-7)
Supplement: Supplementary file 3 — Description of additional supplementary files [file 42003_2021_2482_MOESM3_ESM.pdf]

## Description of Additional Supplementary Files

**File name:** Supplementary Data

**Description:** Source data for Supplementary Fig. 3d.
