# Supplementary material for: Plant death caused by inefficient induction of antiviral R-gene-mediated resistance may function as a suicidal population resistance mechanism
Source: Commun Biol. 2021 Aug 9;4:947. doi: 10.1038/s42003-021-02482-7 (PMC8352862; doi:10.1038/s42003-021-02482-7)
Supplement: Supplementary file 5 — Reporting summary [file 42003_2021_2482_MOESM5_ESM.pdf]

nature portfolio

Corresponding author(s): Shuhei Miyashita

Last updated by author(s): Jul 16, 2021

Reporting Summary

Nature Portfolio wishes to improve the reproducibility of the work that we publish. This form provides structure for consistency and transparency in reporting. For further information on Nature Portfolio policies, see our Editorial Policies and the Editorial Policy Checklist.

Statistics

For all statistical analyses, confirm that the following items are present in the figure legend, table legend, main text, or Methods section.

n/a	Confirmed	
☒	☐	The exact sample size (*n*) for each experimental group/condition, given as a discrete number and unit of measurement
☒	☐	A statement on whether measurements were taken from distinct samples or whether the same sample was measured repeatedly
☒	☐	The statistical test(s) used AND whether they are one- or two-sided<br>*Only common tests should be described solely by name; describe more complex techniques in the Methods section.*
☒	☐	A description of all covariates tested
☒	☐	A description of any assumptions or corrections, such as tests of normality and adjustment for multiple comparisons
☐	☒	A full description of the statistical parameters including central tendency (e.g. means) or other basic estimates (e.g. regression coefficient) AND variation (e.g. standard deviation) or associated estimates of uncertainty (e.g. confidence intervals)
☒	☐	For null hypothesis testing, the test statistic (e.g. *F*, *t*, *r*) with confidence intervals, effect sizes, degrees of freedom and *P* value noted<br>*Give P values as exact values whenever suitable.*
☒	☐	For Bayesian analysis, information on the choice of priors and Markov chain Monte Carlo settings
☐	☒	For hierarchical and complex designs, identification of the appropriate level for tests and full reporting of outcomes
☒	☐	Estimates of effect sizes (e.g. Cohen's *d*, Pearson's *r*), indicating how they were calculated

Our web collection on statistics for biologists contains articles on many of the points above.

Software and code

Policy information about availability of computer code

Data collection	Simulations for SHR(+) plants survival were done with the code provided in "Supplementary Information" as Supplementary Text 2 to 5. They are also available at GitHub/Zenodo under the DOI 10.5281/zenodo.5105622
Data analysis	MOI estimation code is provided in "Supplementary Information" as Supplementary Text 1; we confirmed its function on R versions 3.4.1 and 4.0.0. It is also available at GitHub/Zenodo under the DOI 10.5281/zenodo.5105622

For manuscripts utilizing custom algorithms or software that are central to the research but not yet described in published literature, software must be made available to editors and reviewers. We strongly encourage code deposition in a community repository (e.g. GitHub). See the Nature Portfolio guidelines for submitting code & software for further information.

Data

Policy information about availability of data

All manuscripts must include a data availability statement. This statement should provide the following information, where applicable:

- Accession codes, unique identifiers, or web links for publicly available datasets
- A description of any restrictions on data availability
- For clinical datasets or third party data, please ensure that the statement adheres to our policy

We have added data availability statement. Specifically, sequences of cDNA constructs are deposited in DDBJ/EMBL/GenBank; raw data for images are provided as a supplementary "Supplementary_Data.xlsx" file; all the scripts are provided in "Supplementary Information" file as well as at GitHub/Zenodo under the DOI 10.5281/zenodo.5105622

Field-specific reporting

Please select the one below that is the best fit for your research. If you are not sure, read the appropriate sections before making your selection.

☒ Life sciences ☐ Behavioural & social sciences ☐ Ecological, evolutionary & environmental sciences

For a reference copy of the document with all sections, see nature.com/documents/nr-reporting-summary-flat.pdf

Life sciences study design

All studies must disclose on these points even when the disclosure is negative.

Sample size	For MOI estimation, we know a relation that analyzing 4-times more cells will give half standard deviation for MOI. Thus, we could calculate the number of cells need to be analyzed to have less than 10% of standard deviation to the MOI estimates. As the number of infected sites per a inoculated leaf fluctuates to some extent, we inoculated the plants on several independent days until we have enough number of cells.
Data exclusions	For MOI estimation, we aimed to analyze sites which have cells infected ate the initial infection (Cell-0), after first cell-to-cell movement (Cell-1), and second cell-tocell movement (Cell-2). Our observation showed that the number of Cell-1 and Cell-2 cells are in average ~8 and ~20 per infected site, respectively, thus we excluded the sites with less than 10 infected cells (i.e., not having any Cell-2 cells) and those with more than 29 infected cells (i.e., having Cell-3 cells that are infected after three times of cell-to-cell movements.)
Replication	For MOI estimation of each plant-virus combination, we inoculated independent plants on independent days. We estimated MOI and its standard deviation for each day, and confirmed that estimated MOI of a certain day appears within the standard deviation of the other day(s); this confirms that the MOI estimates of independent days are not significantly different. After that we combined those data of independent experiments and obtained an MOI estimate that appear in the manuscript.
Randomization	No randomization was required for the experiments.
Blinding	No blinding was required for the experiments.

Reporting for specific materials, systems and methods

We require information from authors about some types of materials, experimental systems and methods used in many studies. Here, indicate whether each material, system or method listed is relevant to your study. If you are not sure if a list item applies to your research, read the appropriate section before selecting a response.

Materials & experimental systems

n/a	Involved in the study	
☐	☒	Antibodies
☒	☐	Eukaryotic cell lines
☒	☐	Palaeontology and archaeology
☒	☐	Animals and other organisms
☒	☐	Human research participants
☒	☐	Clinical data
☒	☐	Dual use research of concern

Methods

n/a	Involved in the study	
☒	☐	ChIP-seq
☒	☐	Flow cytometry
☒	☐	MRI-based neuroimaging

Antibodies

Antibodies used	anti-CMV CP antibody was used but was raised in-house and is not commercial.
Validation	The above antibody was validaded in our previous studies, e.g., Takahashi et al. (2012).
